# Comparative transcriptome analysis of compatible and incompatible *Brassica napus*—*Xanthomonas campestris* interactions

**DOI:** 10.3389/fpls.2022.960874

**Published:** 2022-08-29

**Authors:** Li Yang, Chuanji Zhao, Zetao Bai, Lingli Yang, M. Eric Schranz, Shengyi Liu, Klaas Bouwmeester

**Affiliations:** ^1^Biosystematics Group, Wageningen University and Research, Wageningen, Netherlands; ^2^Key Laboratory of Biology and Genetic Improvement of Oil Crops, Ministry of Agriculture, Oil Crops Research Institute, Chinese Academy of Agricultural Sciences, Wuhan, China

**Keywords:** *Brassica napus*, black rot resistance, RNA-seq, differential gene expression, *Xanthomonas campestris* pv. *campestris*

## Abstract

Black rot caused by the vascular pathogenic bacterium *Xanthomonas campestris* pv. *campestris* (*Xcc*) is widespread in Brassicaceae plants and an infectious disease that causes large yield losses in oil seed rape (*Brassica napus* L.). Improvement of resistance through breeding is a crucial strategy to prevent black rot disease in *B. napus*, but presently hampered by insufficient understanding of *Xcc-*Brassica interactions. This study compares two EMS-mutagenized *B. napus* lines that show contrasting resistance levels to their susceptible progenitor. Patterns of differential gene expression between these *B. napus* lines were evaluated at three time points post inoculation by comparative RNA-seq analysis. In line with the observed disease phenotypes, the susceptible line ZS9m*Xcc*S-1 displayed a steady amount of differentially expressed genes (DEGs) at different time points of infection, whereas the resistant line ZS9m*Xcc*R-1 displayed a gradual increase in DEGs throughout the course of infection. Weighted gene co-expression network analysis (WGCNA) pinpointed multiple defense-related hub genes with potential central roles in immunity, including the cell surface receptor genes *CRK11* and *BIR1*, and the associated downstream regulatory genes *WRKY11* and *PBL30*. KEGG analysis of DEGs belonging to two distinct co-expression modules revealed enriched pathways associated with defense, including Ca^2+^-signaling, receptor-mediated immunity, and phytohormone balance. Taken together, our comparative transcriptome analysis provides new avenues to unravel the mechanisms underlying black rot resistance in *B. napus*.

## Introduction

Black rot caused by bacterial pathogen *Xanthomonas campestris* pv. *campestris* (*Xcc*) is one of the most destructive diseases in Brassica crops, including oil seed rape *Brassica napus* L. (Vicente and Holub, 2013). Typical black rot symptoms are V-shaped chlorotic lesions and blackening of leaf veins, which can lead to considerable yield losses—especially when infection occurs under favorable environmental conditions (Aires et al., 2011; Afrin et al., 2018). Currently grown cultivars do not provide adequate black rot resistance, and chemical control is not effective and hazardous to the environment (Vicente and Holub, 2013). As such, Brassica breeding for black rot resistance is considered one of the most effective and sustainable control methods.

Plants are subject to infection by a wide range of pathogens, including diverse pathogenic bacteria. Natural selection has driven plants to shape a sophisticated defense system to resist the attack of pathogenic microbes (Fu and Dong, 2013). Besides constitutive defenses, including structural barriers such as waxy epidermal cuticles and trichomes, plants evolved a multi-layered induced defense system to ward off pathogens. At the cell surface, pattern-recognition receptors (PRRs), either receptor-like kinases (RLKs) or receptor-like proteins (RLPs), recognize pathogen attack by perceiving diverse pathogen-associated molecular patterns (PAMPs). PAMP-triggered immunity (PTI) initiates a suite of downstream immune responses to prevent pathogen invasion, including accumulation of reactive oxygen species (ROS), induction of defense gene expression, Ca^2+^ influx, and plant hormone synthesis (Zipfel, 2014). Plant pathogens are capable to secrete effectors into host cells to suppress PTI by disrupting defense-associated signaling (Dodds and Rathjen, 2010). In turn, plants can detect these effectors or their activity, by intracellular resistance proteins composed of nucleotide−binding and leucine−rich repeat (NLR) domains. Effector-triggered immunity (ETI) generally leads to stronger and more damaging defense reactions, often manifested by a hypersensitive response (HR) leading to host cell death (Hatsugai et al., 2017). Recent studies show that PTI and ETI are interconnected and cross-potentiating each other, which is required to mount robust disease resistance (Ngou et al., 2021; Yuan et al., 2021).

Various studies have been performed to unravel the complexity of Brassica-*Xcc* interactions using QTL mapping and diverse omics approaches (Shaw et al., 2021). Diverse reports revealed correlations between black rot disease severity in Brassica and the accumulation of secondary metabolites, including glucosinolates, hydroxycinnamic acids (HAs), flavonoids, and various phenolics (Aires et al., 2011; Velasco et al., 2013; Barman et al., 2015; Islam et al., 2018). Such defensive metabolites were especially found to accumulate in incompatible interactions (Islam et al., 2017). Resistant *B. napus* cultivars also display high JA levels and enhanced expression of synthesis-related genes upon infection, eventually leading to the accumulation of JA-mediated metabolites functioning in host immunity. The opposite pattern was observed in susceptible cultivars that show enhanced levels of SA and ABA (Islam et al., 2019). Recent transcriptome analysis of two *B. rapa* cultivars with contrasting resistance levels identified the top 10 most differentially expressed genes (DEGs) upon *Xcc* infection, one of which encodes a NLR receptor protein (Song et al., 2020). Similar work by Sun et al. (2021), who characterized early transcriptional defense responses in *B. oleracea*, highlighted the importance of genes involved in the glucosinolate pathway, ROS scavenging, and photosynthetic metabolism in *Xcc* resistance. Despite recent achievements, our present understanding of the molecular mechanisms of black rot resistance in Brassica crops, especially oilseed rape, is still far from complete.

In this study, two ethyl methanesulfonate (EMS)-induced *B. napus* mutant lines representing contrasting levels of black rot resistance—one showing enhanced resistance (ZS9m*Xcc*R-1), the other enhanced susceptibility (ZS9m*Xcc*S-1), were compared with their progenitor *B. napus* ZS9. Whole transcriptome profile analyses were performed with these three lines at different time points of *Xcc* infection. Transcriptional dynamics between different lines post *Xcc* inoculation, such as the differences in GO enrichment and KEGG pathways, were systematically characterized. Candidate genes potentially playing key roles in resistance toward *Xcc* in *B. napus* were pinpointed. Overall, this study provides new insights into the molecular mechanisms underlying black rot resistance in *B. napus* and forms a valuable resource for future functional studies to inform oil seed resistance breeding.

## Materials and methods

### Plant material and growth conditions

This study makes use of *B. napus* cv. Zhongshuang 9 (ZS9), a widely cultivated semi-winter variety with improved traits, including *Sclerotinia* resistance and high yield (Wang et al., 2004). A set of 41 mutants was obtained by ethyl methanesulfonate mutagenesis (EMS) treatment using ZS9 as progenitor. Plant material was grown in a greenhouse under standard conditions [22/18°C (day/night), 16/8 h photoperiod, and > 60–70% relative humidity].

### Inoculum preparation and plant infection assays

*Xcc* strains were grown on plates containing NYGA medium (5 g/L peptone, 3 g/L yeast extract, 20 g/L glycerol, 1.5% agar, pH 7.0) at 28°C for 48 h. Fresh bacterial cultures were dissolved in sterilized ddH_2_O and adjusted to an OD_600_ value of 0.4. Plants at a four-leaf stage were used in disease assays. Three leaves per plant were inoculated by clipping leaf tips (approximately 1–1.5 cm) with scissors dipped in bacterial suspension. Mock control leaves were clipped using scissors dipped in water. After inoculation, greenhouse conditions were set to facilitate *Xcc* infection [28/24°C (day/night), 14/10 h photoperiod, and > 90% relative humidity] (Lema et al., 2012).

### Isolation of pathogenic bacteria from leaf tissue

Leaf disks (5 mm in diameter) sampled at inoculation sites or at the margin of lesions were surface sterilized according to Yang et al. (2019). Samples were grinded using a sterilized microcentrifuge pestle, and finally left to set for 15 min. Suspensions were diluted 100 times, of which 50 μl was plated on NYGA plates and incubated at 28°C. Experiments were conducted with three biological repetitions.

### Tissue sampling and RNA isolation

At 5 and 8 days post inoculation (dpi), lesion areas including a margin of seemingly unaffected tissue were sampled using surgical scissors from each inoculated third true leaf. Leaf tissue of mock-inoculated plants was sampled approximately 5 mm from the point of inoculation. Samples were flash-frozen in liquid nitrogen and stored at –80°C. Total RNA was isolated using a TGuide plant RNA extraction kit (TIANGEN, Bei Jing, China) following the manufacturer’s instructions. RNA was purified by ethanol precipitation. Concentration and purity of RNA samples was measured with a NanoDrop 2000c spectrophotometer and a Qubit 4.0 fluorometer using the RNA Broad Range assay (Thermo Fisher Scientific, United States). Experiments were performed with three biological repetitions.

### Comparative transcriptome analysis

Library preparation and RNA sequencing was performed at the Novogene Bioinformatics Institute (Beijing, China) according to Yang et al. (2018). Adapter sequences and low quality sequence reads were filtered from datasets using the NGSQC Toolkit (Patel and Jain, 2012). Clean reads were mapped to the *B. napus* reference genome *Darmor-bzh* by Hisat2 v2.0.5 (Chalhoub et al., 2014; Kim et al., 2015). Transcripts were quantified by stringtie v1.3.3b as Fragments Per Kilobase of exon model per million mapped reads (FPKM) (Pertea et al., 2015). Genes with expression differences meeting the thresholds |log2(FoldChange)| > 1 and padj < 0.05 were designated as DEGs by DESeq2 (Love et al., 2014). Principle component analysis (PCA) was conducted with the princomp function, and subsequent correlation analysis was performed using the corrplot package in R. K-means clustering was analyzed based on normalized FPKM by Genesis software (Sturn et al., 2002). Raw data of RNA-seq was available from the NCRI SRA database (PRJNA748871).

### Functional annotation and enrichment analysis of differentially expressed genes

Functional annotation of DEGs was performed by similarity searches against the TAIR10 and NCBI non-redundant (nr) protein databases using blastx with an *e*-value of 10^–5^ as cut-off. GO enrichment analysis was implemented by clusterProfiler package in R (Yu et al., 2012). GO terms with a padj value < 0.05 were assigned as significantly enriched. KEGG pathways involved in these DEGs were analyzed using the webserver KOBAS v2.0 with *p* < 0.05 as cut-off criterion (Xie et al., 2011).

### Gene co-expression network analysis

Weighted gene co-expression network analysis was implemented with the WGCNA package in R (Langfelder and Horvath, 2008; Supplementary Data 1). DEGs were preliminarily filtered using the FPKM threshold of 300 over all samples. Data clustering was performed to detect outliers. Weighted networks with scale-free topology were constructed using a soft thresholding power of β = 18 with a *R*^2^-value > 0.85. Obtained modules were color-coded, among which the gray module contains genes not belonging to any of the other modules. Heatmap and module clustering were used to visualize the co-expression network of 1,000 randomly selected genes. Color intensity represents the level of expression similarity among genes. Expression levels of DEGs within each module were depicted in a heatmap plot, and corresponding GO and KEGG enrichment analyses were performed as described above. Correlations between different modules and conditions were calculated to pinpoint the most correlated module-trait relationships. Selected modules were finally visualized by VisANT software (Hu et al., 2013).

### Quantitative RT-PCR

Synthesis of *B. napus* cDNA was conducted using a PrimeScript RT reagent Kit with gDNA Eraser according to the manufacturer’s instructions. *BnActin* (GenBank: AF111812.1) was used as the reference gene to normalize and quantify target gene expression using the 2^–ΔΔCt^ method (Livak and Schmittgen, 2001). Q-RT-PCR reactions were performed on a CFX Connect Real-time PCR system (Bio-Rad, United States) using SYBR Green Real-time PCR Master Mix. Relative expression levels are given as mean values ± standard deviation (SD) based on three biological repetitions. Gene-specific primers for Q-RT-PCR were designed with the online tool Primer3Plus and listed in Supplementary Table 1.

## Results

### Two ethyl methanesulfonate-mutagenized *Brassica napus* lines show contrasting phenotypes to black rot disease

**FIGURE 1:**
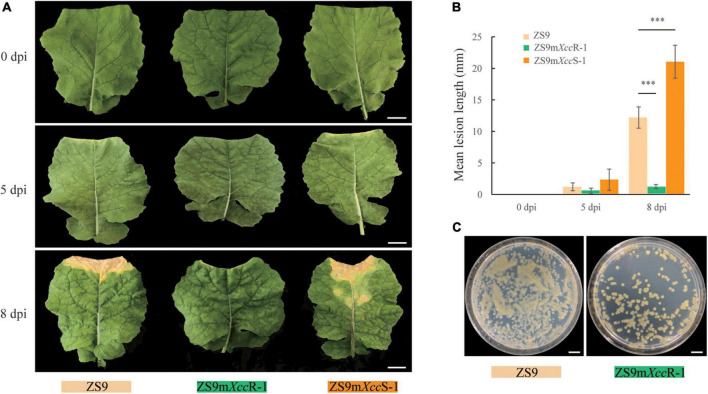
Two *B. napus* EMS lines showing contrasting levels of *Xcc* resistance. **(A)** Disease symptoms observed on the parental line ZS9 and the two EMS lines ZS9m*Xcc*R-1 and ZS9m*Xcc*S-1. Pictures were taken at 0, 5, and 8 days after *Xcc* inoculation. **(B)** Lesion lengths on ZS9, ZS9m*Xcc*R-1, and ZS9m*Xcc*S-1 after inoculation with *Xcc*. Bars represent mean lesion lengths ± SD based on three biological repetitions. ^***^ indicate significant differences (*p* < 0.001) according to Student’s *t*-tests. **(C)** Extracts from ZS9m*Xcc*R-1 showing reduced bacterial titers compared to ZS9. Bars represent 20 mm in **(A)** and 10 mm in **(C)**.

A set of 41 EMS-mutagenized *B. napus* lines derived from accession ZS9 (TH26) was screened for disease resistance to *Xcc* race 3 (Supplementary Figure 1). Two lines showed contrasting phenotypes in resistance compared to its susceptible progenitor. TH316 displayed a strong resistance to race 3, whereas TH315 showed an enhanced disease susceptibility. These lines were hereafter named ZS9m*Xcc*R-1 and ZS9m*Xcc*S-1, respectively. To further evaluate and characterize this differential performance, additional disease assays were conducted on these three lines using lesion length and bacterial growth as measures of disease progression (Figure 1). Disease symptoms started to appear on ZS9m*Xcc*S-1 and ZS9 5 days after inoculation with *Xcc* race3. In contrast, no visual symptoms could be detected on inoculated ZS9m*Xcc*R-1 plant. At 8 dpi, clear V-shaped lesions could be observed on leaves of both ZS9 and ZS9m*Xcc*S-1 with mean lengths of 10 and 20 mm, respectively. Again, no lesions were found on ZS9m*Xcc*R-1 leaves, displaying only minor browning at the zone of inoculation (Figures 1A,B). In addition, leaf disks sampled from lesions on ZS9 plants were found to contain high bacterial numbers. Inoculated ZS9m*Xcc*R-1 leaves only contained few bacteria, underlining the observed resistance to race 3 of *B. napus* ZS9m*Xcc*R-1 (Figure 1C). To assess whether ZS9m*Xcc*R-1 displays broad-spectrum resistance to black rot, we performed diseases assays with different *Xcc* races. This revealed that ZS9m*Xcc*R-1 not only shows resistance to *Xcc* race 3, but also displays gain of resistance to races 2 and 4, but not to races 1, 6, and *Xcc*-15. In contrast, both ZS9 and ZS9m*Xcc*S-1 were found to be susceptible to all tested races (Supplementary Figure 2).

### Comparative transcriptome profile analyses of two ethyl methanesulfonate mutants and the parental line Zhongshuang 9

To examine transcriptional dynamics upon *Xcc* infection in *B. napus*, RNA-seq analysis was performed on leaves of ZS9, ZS9m*Xcc*R-1, and ZS9m*Xcc*S-1 at 0, 5, and 8 dpi. Each sampling point contained three biological replicates, resulting in 27 libraries (Supplementary Table 2). A total of 1.2 billion raw reads was generated, with approximately 45 million reads per library. Clean reads after filtering were mapped to the *B. napus* reference genome *Darmor-bzh* (Chalhoub et al., 2014). The percentage of mapped reads was found to decrease gradually in the susceptible lines ZS9 and ZS9m*Xcc*S-1 during the time course of experiment. This observation is in line with the biological composition of the samples, in which the amount of *Xanthomonas* bacteria in the plant tissue increases over time. Detailed information on the quality of sequence reads, GC content, and the ratios of uniquely mapped reads are summarized in Supplementary Table 2.

**FIGURE 2:**
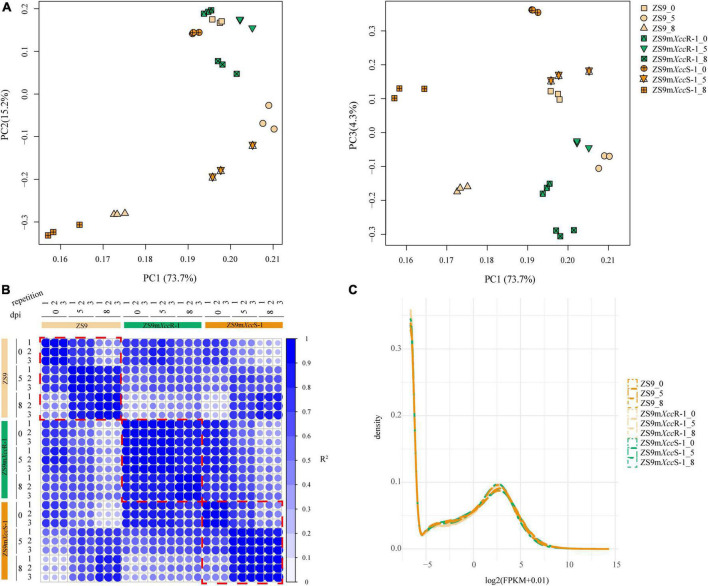
Quality evaluation of RNA-seq data. **(A)** PCA displaying the major composition of variance. Left panel: 73.7 and 15.2% of the variance referred to PC1 and PC2, respectively. Right panel: 73.7 and 4.3% of the variance referred to PC1 and PC3, respectively. **(B)** Representation of correlation matrix between RNA-seq samples based on Pearson correlation coefficients. Numbers represent biological repetitions. **(C)** Frequency histogram representing the distribution of gene expression levels across samples. Numbers 0, 5, and 8 indicate days post inoculation.

To provide a comprehensive overview of the transcriptome data and to exclude noise and redundancy effects, a principal component analysis (PCA) was conducted based on gene read counts (Figure 2A). Replicates per treatment were found to cluster together both in PC1/2 and PC1/3. Subsequent, correlation analysis of FPKM values showed high correlation coefficients between different repetitions per treatment (Figure 2B). Additionally, the density distribution of gene expression levels showed a consistent tendency across different samples (Figure 2C). These results indicated high reproducibility of RNA-seq data allowing further in-depth analyses.

### Transcriptional dynamics in *Brassica napus* after *Xanthomonas campestris* pv. *campestris* inoculation

**FIGURE 3:**
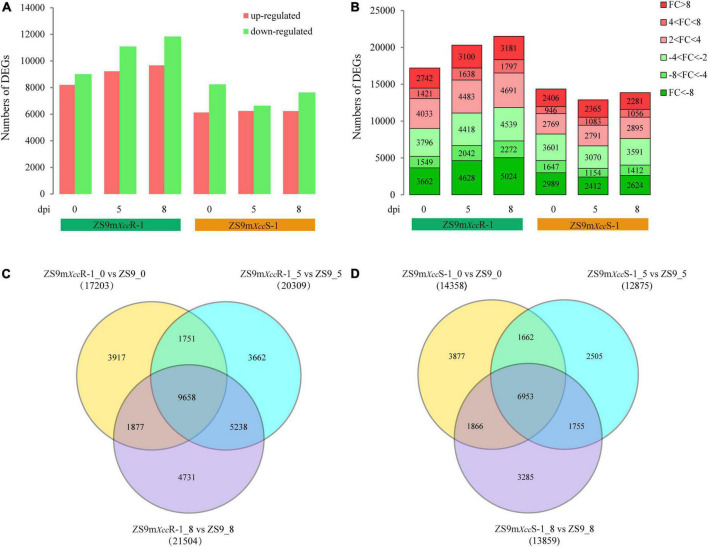
Comparative analysis of differentially expressed genes between *B. napus* ZS9 and the two EMS-lines ZS9m*Xcc*R-1 and ZS9m*Xcc*S-1 at different time points post inoculation. **(A)** Numbers of up-regulated and down-regulated DEGs in ZS9m*Xcc*R-1 and ZS9m*Xcc*S-1 at 0, 5, and 8 dpi. **(B)** Fold change (FC) distribution of DEGs in ZS9m*Xcc*R-1 and ZS9m*Xcc*S-1 at 0, 5, and 8 dpi. FC was calculated as the ratio of gene read counts between ZS9m*Xcc*R-1/ZS9m*Xcc*S-1 and ZS9. Venn diagrams displaying numbers of common and unique DEGs in the resistant ZS9m*Xcc*R-1 **(C)** and the susceptible ZS9m*Xcc*S-1 **(D)** at three time points post inoculation, respectively.

To investigate expression changes in *B. napus* lines with different degrees of susceptibility to *Xcc*, we compared RNA-seq data of the EMS-mutant lines with that of the ZS9 parental line at same sampling time. This resulted in the comparison of six data pairs (ZS9m*Xcc*R-1_0 vs. ZS9_0, ZS9m*Xcc*R-1_5 vs. ZS9_5, ZS9m*Xcc*R-1_8 vs. ZS9_8, ZS9m*Xcc*S-1_0 vs. ZS9_0, ZS9m*Xcc*S-1_5 vs. ZS9_5, and ZS9m*Xcc*S-1_8 vs. ZS9_8). DEGs were defined as genes that were significantly different in at least one comparison pair after integrating all transcriptome profiles. In total, 37,744 DEGs were determined (Supplementary Table 3). The numbers of up-regulated and down-regulated DEGs detected at different time points in each *B. napus* line were counted and visualized in a bar plot (Figure 3A). Remarkably, ZS9m*Xcc*R-1 had both more up-regulated and down-regulated DEGs at every sampling time compared to ZS9m*Xcc*S-1, implying that the difference between ZS9m*Xcc*R-1 and ZS9 is larger than that between the susceptible lines ZS9m*Xcc*S-1 and ZS9. DEGs grouped according to their fold changes (FC) were found to be unevenly distributed across all samples, most of which had absolute FC values between 2 and 4 or higher than 8 (Figure 3B).

Comparative analysis of DEGs between ZS9m*Xcc*R-1 and ZS9 after *Xcc* inoculation showed strong overlap, in which almost half of the total DEGs were overlapping between the three sampling points (Figure 3C). This also accounted for the paired comparison of ZS9m*Xcc*S-1 and ZS9, which had 6,953 DEGs in common (Figure 3D). Notably, the total number of DEGs expressed at 5 and 8 dpi in ZS9m*Xcc*R-1 vs. ZS9 (5,238) was found to be almost three times higher as within the ZS9m*Xcc*S-1 vs. ZS9 comparison (1,755). These results revealed that the resistant line ZS9m*Xcc*R-1 and susceptible ZS9m*Xcc*S-1 show the most distinct patterns of gene expression upon *Xcc* infection.

**FIGURE 4:**
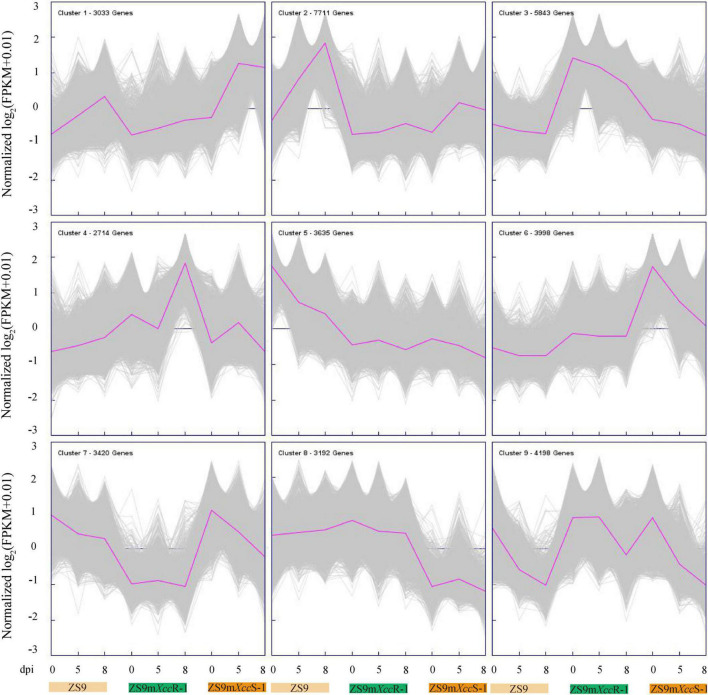
K-means cluster analysis of 37,744 DEGs. Purple lines indicate average expression levels of the subcluster. Gray lines represent expression levels of each gene within the cluster. The number of genes per cluster is indicated at the left corner of each panel.

To further differentiate DEGs in response to *Xcc* inoculation, K-means clustering was implemented to classify 37,744 genes based on their expression patterns, resulting into nine different clusters (Figure 4). Genes in cluster 1 display a rather stable expression in ZS9m*Xcc*R-1, but were found to be up-regulated at 5 dpi in ZS9m*Xcc*S-1 and at both 5 and 8 dpi in ZS9. Cluster 2 shows a quite similar pattern with genes that have higher expression levels in ZS9 at 8 dpi. Most genes grouped in clusters 3 and 9 were found to be down-regulated at all-time points in the three *B. napus* lines, except for DEGs in ZS9m*Xcc*R-1 at 5 dpi within cluster 9. This down-regulation of gene expression within the susceptible lines ZS9m*Xcc*S-1 and ZS9 was also detected in clusters 6 and 7, and clusters 5 and 7, respectively. Cluster 4 contains genes moderately down-regulated in ZS9m*Xcc*R-1 at 5 dpi and significantly up-regulated at 8 dpi, while the expression tendency in ZS9m*Xcc*S-1 was exactly the opposite, supplying valuable clues to understand the contrasting phenotypes of these *B. napus* lines in *Xcc* resistance.

### Functional enrichment and comparative analysis of differentially expressed genes

**FIGURE 5:**
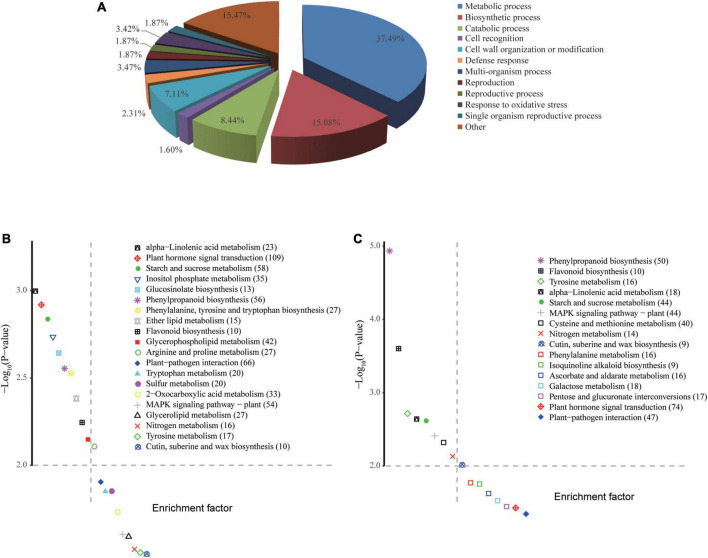
GO enrichment and KEGG pathway analyses of DEGs. **(A)** Biological classification of all DEGs by GO enrichment analysis. **(B,C)** KEGG pathways enriched in ZS9m*Xcc*R-1 and ZS9m*Xcc*S-1, respectively. The vertical and horizontal axes of KEGG scatter diagrams represent the –log_10_(*P*-value) and enrichment factor, respectively. Values in brackets are indicative of the number of DEGs enriched in each pathway.

Identified DEGs were functionally annotated by performing blastx against the TAIR10 and NCBI nr protein databases. Subsequently, these DEGs were subjected to functional GO enrichment analysis using the clusterProfiler package in R. With a threshold cutoff of padj < 0.05, DEGs could be clustered into 106 GO groups divided over the three functional categories (Supplementary Table 4). Most significantly enriched GO terms of DEGs were related to biosynthetic processes, such as the metabolism of polysaccharides (GO:0005976) and glucans (GO:0006073, GO:0044042), and cell wall organization (e.g., GO:00071554). GO terms related to oxidative stress, defense response, and cell wall modification also made up a considerable proportion, underlining the biotic stress conditions evoked in *B. napus* upon *Xcc* infection (Figure 5A). Enrichment analysis revealed that DEGs expressed in the resistant line ZS9m*Xcc*R-1 were mainly involved in cellular metabolic processes, cell wall organization and modification, cell recognition, and the response to oxidative stress (Table 1 and Supplementary Figure 3A). As for the susceptible line ZS9m*Xcc*S-1, unique GO terms of DEGs were found to be related to defense response, reaction to inorganic substances, and the carbohydrate metabolic process, but lacked categories involved in cell recognition, and cell wall organization and modification as enriched in the resistant line (Table 1 and Supplementary Figure 3B).

**TABLE 1:** Comparison of GO-terms (biological processes) enriched in ZS9m*Xcc*R-1 and ZS9m*Xcc*S-1 upon *Xcc* inoculation.

		ZS9m*Xcc*R-1/ZS9		ZS9m*Xcc*S-1/ZS9	
GO_ID	Term	n	–Log (padj)	n	–Log (padj)
**Stress response**					
GO:0006979	Response to oxidative stress	121	2.64	96	3.04
GO:0006952	Defense response	–	–	65	2.07
GO:0010035	Response to inorganic substance	–	–	23	1.78
**Metabolism synthesis**					
GO:0044262	Cellular carbohydrate metabolic process	198	5.51	165	9.14
GO:0006022	Aminoglycan metabolic process	40	5.46	30	3.04
GO:0006030	Chitin metabolic process	40	5.46	30	3.04
GO:0006040	Amino sugar metabolic process	40	5.46	30	3.04
GO:0044036	Cell wall macromolecule metabolic process	40	5.46	30	3.04
GO:1901071	Glucosamine-containing compound metabolic process	40	5.46	30	3.04
GO:0006073	Cellular glucan metabolic process	123	4.20	103	6.13
GO:0044042	Glucan metabolic process	123	4.20	103	6.13
GO:0044264	Cellular polysaccharide metabolic process	123	4.20	103	6.13
GO:0005976	Polysaccharide metabolic process	139	4.08	117	6.18
GO:0006020	Inositol metabolic process	16	1.75	15	2.76
GO:0044723	Single-organism carbohydrate metabolic process	–	–	176	1.78
GO:0005991	Trehalose metabolic process	–	–	28	1.54
GO:1901615	Organic hydroxy compound metabolic process	–	–	25	1.32
GO:0051273	Beta-glucan metabolic process	–	–	55	1.32
**Cell**					
GO:0071554	Cell wall organization or biogenesis	123	5.51	92	3.89
GO:0016998	Cell wall macromolecule catabolic process	40	5.46	30	3.04
GO:0071555	Cell wall organization	73	2.52	–	–
GO:0008037	Cell recognition	57	2.03	–	–
GO:0034637	Cellular carbohydrate biosynthetic process	110	1.94	89	3.04
GO:0042545	Cell wall modification	56	1.92	–	–
GO:0044275	Cellular carbohydrate catabolic process	–	–	9	1.78

KEGG enrichment analyses were implemented to determine pathways associated with the identified DEGs. Comparing ZS9m*Xcc*R-1 with ZS9 resulted in the identification of 20 significant enriched KEGG pathways, whereas the pair-wise comparison of ZS9m*Xcc*S-1 and ZS9 resulted in 16 pathways (Figures 5B,C). Abundant pathways associated with plant defense were commonly detected in both ZS9m*Xcc*R-1 and ZS9m*Xcc*S-1 lines following *Xcc* inoculation, such as those involved in sucrose metabolism, phenylpropanoid biosynthesis, and plant-pathogen interactions. Pathways specifically assigned to the resistant line ZS9m*Xcc*R-1 were related to lipid metabolism and the biosynthesis of glucosinolates. Pathways unique to the susceptible line ZS9m*Xcc*S-1 were associated with metabolism of galactose, ascorbate, and various amino acids (Figures 5B,C).

### Co-expression network analysis identifies hub genes associated with black rot resistance

**FIGURE 6:**
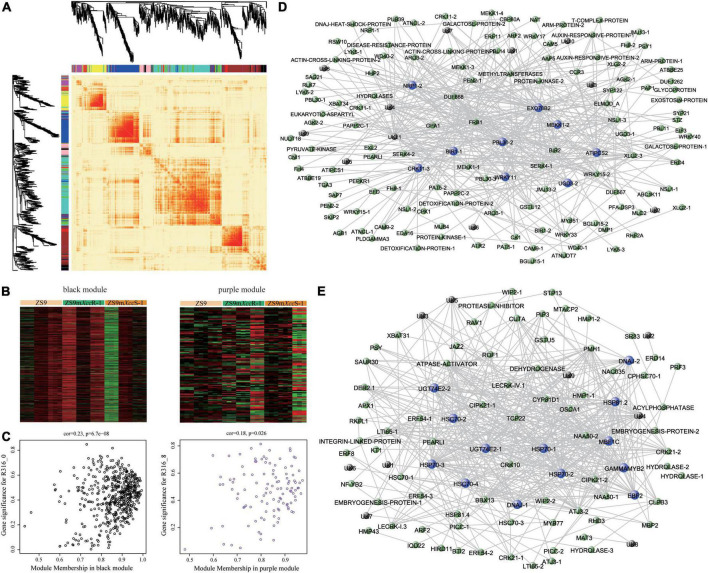
Weighted gene co-expression network analysis identifies hub genes functioning in *Xcc* resistance. **(A)** Heatmap plot showing patterns of differential gene expression across modules. Colored bars below dendrograms indicate respective modules. Color intensity represents the level of expression similarity among genes. **(B)** Expression heatmaps of DEGs clustered in black (left) and purple (right) modules. **(C)** Scatter plots displaying correlation relationships between gene-modules and gene-traits. Weighted gene co-expression networks of the black **(D)** and purple **(E)** modules. Highly connected hub genes are indicated by blue nodes. Black nodes represent genes of unknown function.

To further mine potential genes related to black rot resistance, a weighted gene co-expression network analysis (WGCNA) was implemented using filtered DEG expression levels. A scale-free network was constructed with the soft threshold power of 18 under the threshold *R*^2^ > 0.85, resulting in 11 modules (Supplementary Figure 4A). Gene expression heatmaps and tree diagrams were used to represent a similarity matrix of gene co-expression. DEGs belonging to individual modules showed strong co-expression patterns, especially in the blue, turquoise, and brown modules (Figure 6A). Heatmap visualization of DEGs and GO functional enrichment classifications were performed for each individual module (Figure 6B, Supplementary Figure 4B, and Supplementary Table 5). Correlation analysis of module-treatment relationships showed that genes in black and purple modules are strongly associated with disease severity (ZS9m*Xcc*R-1_0, *r* = 0.49, *P* = 0.01; ZS9m*Xcc*R-1_8, *r* = 0.56, *P* = 0.002) (Supplementary Figure 4C). In addition, we also noticed that these DEGs were not only correlated with their corresponding modules, but also with their corresponding traits (Figure 6C), highlighting the significance to further study the genes within these modules. Genes showing this expression pattern potentially can explain the underlying mechanisms of black rot resistance. DEGs of the black module were found to be up- and down-regulated at 8 dpi in the resistant and susceptible EMS lines, respectively (Figure 6B). Network visualization revealed highly interconnected signatures of gene co-expression within the black module, identifying several defense-associated hub genes—including the cell surface receptors BIR1 and CRK11, the downstream regulatory proteins PBL30 and MEKK1, and the transcription factor WRKY11. Other hub genes with enhanced expression levels in the resistant line are encoding the exocyst complex protein EXO70B2, a Development and Cell Death (DCD)-domain containing protein, an inositol phosphorylceramide synthase, and an UDP-glucose 6-dehydrogenase (Figure 6D and Supplementary Figure 4D). In contrast, DEGs belonging to the purple module were found to be up-regulated in ZS9m*Xcc*R-1 and down-regulated in ZS9m*Xcc*S-1, or vice versa, during the course of infection. Co-expression analysis indicated that the highly interconnected genes were mainly involved in stress tolerance, including a Myb-like HTH transcription factor and multiple heat shock proteins. Two other hub genes within this cluster showing enhanced expression only in the resistant line are encoding the immune-related EIN3-binding F-box protein 2 and the Uridine diphosphate glycosyltransferase 74E2, which were suggested to play roles in ROS integration and hormone-response pathways (Figure 6E and Supplementary Figure 4D).

### Pathways potentially involved in *Brassica napus* resistance against *Xanthomonas campestris* pv. *campestris*

**FIGURE 7:**
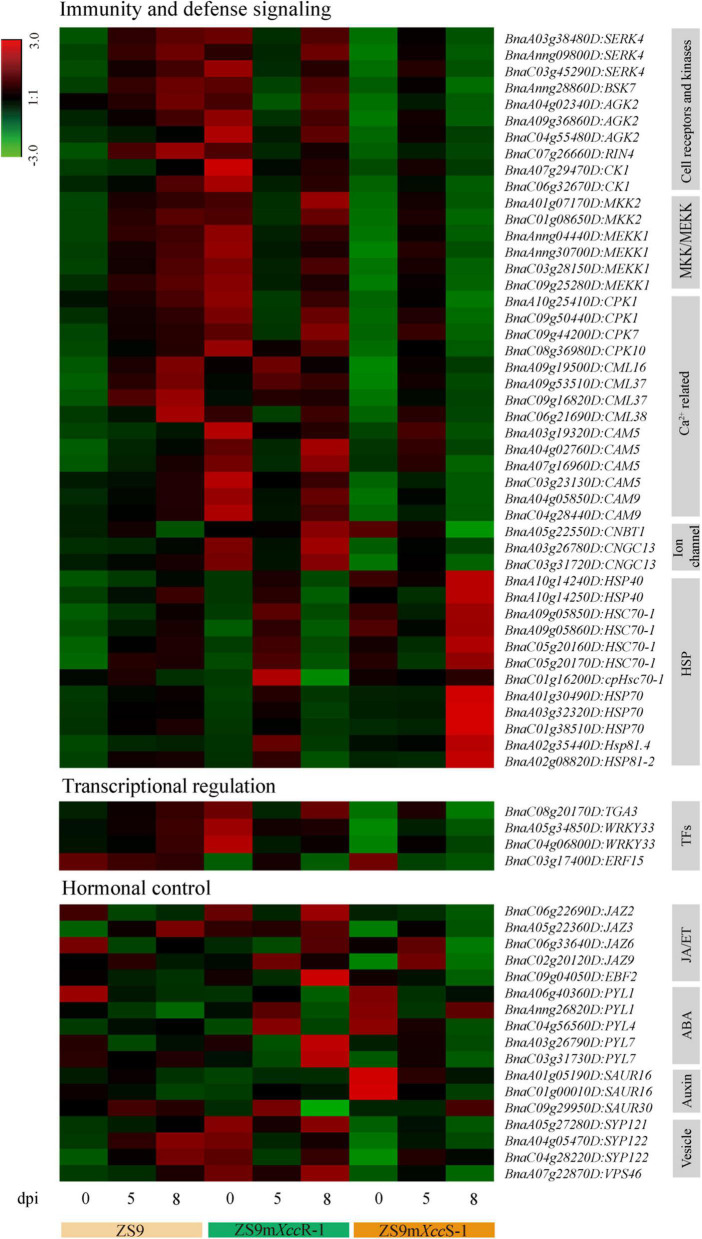
Heat map visualization of selected DEGs related to immunity and defense signaling, transcriptional regulation, and hormonal control.

As a next step, we performed KEGG pathway analyses on DEGs of the black and purple modules. This resulted in the identification of diverse biological pathways involved in black rot resistance, of which several are highlighted below (Figure 7 and Supplementary Table 6).

#### Immunity and defense signaling

A large proportion of DEGs was found to be significantly enriched in pathways related to plant immunity, including components of both immune recognition and downstream signaling—such as the protein kinases SERK4 and BSK7. We also identified multiple copies of *MKK2* and *MEKK1* with differential expression levels in the susceptible and resistant *B. napus* lines. Besides DEGs in these pathways, multiple genes related to calcium (Ca^2+^) signaling, including several calcium-dependent protein kinases (CPKs) and calmodulin proteins (CaMs and CMLs) reported to be involved in immune homeostasis (Choi et al., 2009; Bredow et al., 2021), were identified and shown to be suppressed in expression in the hypersusceptible line at a late phase of infection. Furthermore, we found several cyclic nucleotide-gated ion channels (CNGCs), which are known to function in Ca^2+^ signaling, ROS accumulation, and PTI toward *Xanthomonas oryzae* in rice (Zhang et al., 2018). In contrast, several heat shock protein (HSP)-genes showed high transcript levels in ZS9m*Xcc*S-1 at 8 dpi, reflecting pathogenesis-related stress evoked by *Xcc* proliferation within the leaf tissue (Figure 7).

#### Transcriptional regulation

Transcriptional control of immune-responsive genes is crucial to mount robust immune responses. A multitude of transcription factor genes (TFs) was identified across the full dataset of DEGs, including members of the *MYB*, *bZIP*, and *WRKY* families (Supplementary Table 3). Only a few TFs, however, were found to be enriched in the black and purple modules (Figure 7). Among these, *WRKY33* and *TGA3*—two known host immunity regulators (Wang et al., 2018; Cai et al., 2021)—showed relatively high levels of expression directly after inoculation in ZS9m*Xcc*R-1, but in ZS9m*Xcc*S-1 only at 5 dpi, implying a potential primed state of defense in the resistant line.

#### Hormonal control

Phytohormone signal transduction was also found to be significantly enriched pathway, including DEGs involved in abscisic acid (ABA), auxin, and jasmonic acid/ethylene (JA/ET) signaling. Multiple DEGs were found to encode different orthologs of the JAZ family that function as hormonal crosstalk hubs in JA-mediated plant immunity (Liu et al., 2017). Furthermore, several members of the PYL family that play crucial roles in ABA sensing and environmental stress signaling were detected, showing differential expression patterns during both compatible and incompatible interactions. Two of the three identified auxin-associated *SAUR* genes, known to regulate diverse plant developmental processes, were found to be down-regulated during infection in the hypersusceptible line ZS9m*Xcc*S-1.

### Validating patterns of differential gene expression

**FIGURE 8:**
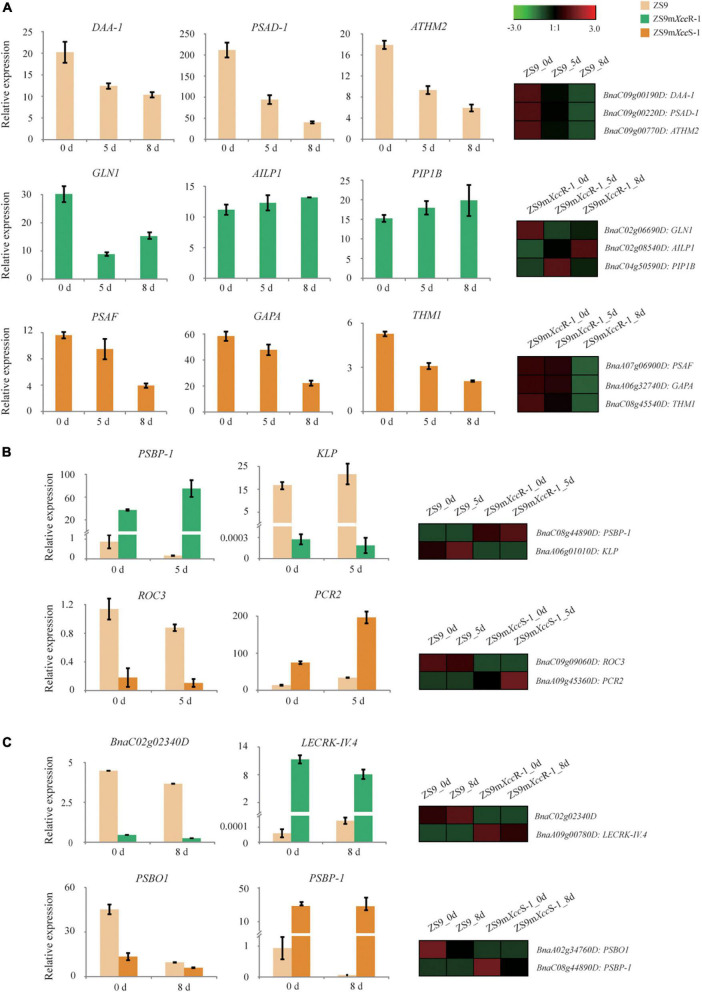
Validation of DEG expression patterns in *B. napus* upon *Xcc* inoculation using Q-RT-PCR. **(A)** Expression levels of representative DEGs with different patterns in ZS9 (top), ZS9m*Xcc*R-1 (middle), and ZS9m*Xcc*S-1 (lower panel) at 0, 5, and 8 dpi, respectively. **(B)** Expression levels of two selected DEGs in ZS9 vs. ZS9*Xcc*R-1 (top) and ZS9 vs. ZS9*Xcc*S-1 (down) at 0 and 5 dpi, respectively. **(C)** Expression levels of two selected DEGs in ZS9 vs. ZS9*Xcc*R-1 (top) and ZS9 vs. ZS9*Xcc*S-1 (down) at 0 and 8 dpi, respectively. Differential expression patterns of selected genes obtained by RNA-seq analysis are shown at the right margin.

To verify our transcriptome data, Q-RT-PCR was performed to validate the transcriptional expression of representative DEGs with different patterns across the biological samples (Supplementary Table 1). As a first step, we randomly selected three DEGs per plant line to verify patterns of differential gene expression. Expression of these selected genes was found to be largely consistent with their transcript profiles obtained by RNA-seq analysis (Figure 8A), such as *DAA-1*, *PSAD-1* and *ATHM2* that display a gradual reduction of gene expression in ZS9 across the time course of infection by both qRT-PCR and RNA-seq. Overall, these results imply strong technical and biological reproducibility between different replicates.

In addition, we ranked DEGs based on levels of fold changes within both paired combination (ZS9m*Xcc*S-1/ZS9 and ZS9m*Xcc*R-1/ZS9), and selected DEGs from the top 50 up- and down-regulated for expression validation. Expression levels of the selected DEGs at 5 and 8 dpi, as measured by Q-RT-PCR, were found to match the patterns of gene expression generated by RNA-seq (Figures 8B,C). For example, *BnaA09g00780D*, an ortholog of the *Arabidopsis* lectin kinase gene *LecRK-IV.4*, was found to be one of most induced genes in the resistant line (Figure 8C). *LecRK-IV.4* was previously shown to play an important role in immune responses to the bacterial pathogen *Pseudomonas syringae* in *Arabidopsis* (Wang et al., 2014). Correlation analysis of gene expression based on Q-RT-PCR and RNA-seq showed significant correlation, demonstrating the reproducibility and accuracy of our RNA-seq data (Supplementary Figure 5).

## Discussion

### Comparative transcriptome analyses provide insights into molecular *Brassica napus-Xanthomonas campestris* pv. *campestris* interactions

Black rot is one of the major vascular diseases in *B. napus* leading to large yield and economic losses. Although considerable efforts have been made to understand genetic mechanisms of black rot resistance in Brassica crops (Islam et al., 2017, 2019; Song et al., 2020; Sun et al., 2021), key genes underlying variation in black rot resistance are thus far largely unknown. Insights into the transcriptional dynamics of plant-pathogen interactions can help to potentially pinpoint genes underlying pathogen resistance (Meng et al., 2021). To elucidate the transcriptional response of *B. napus* to black rot infection, we performed comparative transcriptome sequencing of three *B. napus* lines with different levels of susceptibility at three time points after *Xcc* inoculation. Since Brassica-*Xcc* bioassays are prone to variability (Wei et al., 2016), considerable time and effort was invested to ensure proper biological replication under optimal conditions. This resulted in high correlation between RNA-seq samples, implicating reliability and accuracy of our *B. napus*-*Xcc* bioassays (Figure 2).

In this study, a total of 37,744 DEGs were subjected to subsequent GO and KEGG pathway enrichment analyses after transcript quantification. Gene set pair-wise comparison revealed both shared and unique terms and/or enriched pathways when comparing ZS9m*Xcc*S-1 and ZS9m*Xcc*R-1 with ZS9, respectively. Subsequently, DEGs were categorized into distinct modules based on their co-expression levels using WGCNA analysis, and modules correlating with the phenotypic trait,—including black and purple modules, were further analyzed in detail. We identified several hub genes in these modules with high interconnectivity to the other genes, including multiple cell surface receptors and downstream regulatory proteins involved in immune signaling (Figures 6D,E). Co-expression network and enrichment analyses pinpointed multiple biological processes underlying *Xcc* resistance in *B. napus*, including immunity and defense signaling, transcriptional regulation, and hormonal control (Figure 7).

### Transcriptional insights into *Xanthomonas campestris* pv. *campestris*-induced defense signaling

Our transcriptomic analyses revealed that especially *B. napus* genes involved in early pathogen recognition and subsequent signaling are differentially expressed upon *Xcc* infection. This includes several genes encoding receptor-like kinases (RLKs) and cytoplasmic RLCKs functioning in plant immunity. Cysteine-rich receptor kinases (CRKs), such as CRK11—one of the identified hub genes, are known to play key roles in biotic stress responses and ROS signaling (Little et al., 2006; Bourdais et al., 2015). Overexpression of several *CRKs* in *Arabidopsis* was shown to enhance PTI responsiveness and resistance to *P. syringae* (Acharya et al., 2007; Yeh et al., 2015; Yadeta et al., 2017). Furthermore, several induced *SERK4* genes were found in the resistant line ZS9m*Xcc*R-1. SERK family members are known to function as co-receptors of multiple ligand-induced PRRs functioning in plant immunity, such as FLS2 that mediates PTI by perceiving the bacterial PAMP flagellin (Ma et al., 2016). SERK4 was shown to interact with BAK1 (SERK3) to regulate the stability of the calcium channel CGNC20 to control ROS accumulation and cell death (Yu et al., 2019). Notably, CGNC13, a closely related homolog, was one of the identified DEGs in our study. CNGC1 and CGNC14 have been shown to negatively regulate non-host resistance to *Xanthomonas oryzae* in tomato (Zhang et al., 2018). Another key player in PRR-mediated defense found to be induced in expression upon *Xcc* infection in *B. napus* is the immune repressor gene *BIR1*. Overexpression of *BIR1* in *Arabidopsis* leads to constitutive activation of defense responses, promoting resistance and cell death (Guzmán-Benito et al., 2019). This is in line with the expression pattern observed in the resistant and susceptible lines following *Xcc* inoculation in this study (Supplementary Figure 4D). Another hub gene identified is *PBL30*, a member of the larger RLCK VII subfamily (Rao et al., 2018). PBLs are known as central regulators of both PTI and ETI. One of these, BIK1, associates with the FLS2/BAK1 complex to activate PTI upon perception of bacterial flagellin (Lu et al., 2009). BIK1 is targeted by the *Xcc* effector AvrAC to enhance host susceptibility (Wang et al., 2015). PBL2, a paralog of BIK1, acts as a decoy and triggers ETI upon AvrAC binding via the interaction with ZAR1-mediated resistosome (Wang et al., 2015).

Vesicle-trafficking seems to be another important process during *Xcc* infection, emphasizing the importance of early recognition and defense responses at the host-pathogen interphase. We, for example, detected several closely related syntaxin (*SYP121/122*) genes known to play roles in vesicle-mediated pre- and post-invasive immunity to fungal and oomycete pathogens (Rubiato et al., 2022). *Arabidopsis syp121/syp122* mutants show hypersensitivity to SA and display consecutive expression of multiple immune signaling pathways, both impacting PTI- and ETI-mediated defense (Zhang et al., 2008). Also one exocyst subunit gene, i.e., *EXO70B2*, was found to be differentially expressed in the resistant line. A recent study showed that EXO70B1 and EXO70B2 are required for proper trafficking of FLS2 and potential other immune receptors to the plant plasma membrane (Du et al., 2018; Wang et al., 2020).

Downstream genes involved in immune signaling were also found to be enhanced in expression, mostly in incompatible interactions. For example, multiple mitogen-activated protein kinase (MAPK) cascade genes, including *MKK2* and *MEKK1*, were found relatively highly expressed in ZS9m*Xcc*R-1 at a late stage of infection, and much lower in compatible interactions (ZS9m*Xcc*S-1 and ZS9) (Figure 7). MKK2 and MEKK1 are known to play co-roles in downstream signaling cascades regulating PTI responses (Gao et al., 2008). Recently, MPK1 was shown to promote vascular defense to *Xcc* and *Xoo* in *Arabidopsis* and rice, respectively (Lin et al., 2022). Expression patterns positively correlating with resistance were also found for multiple genes involved in Ca^2+^-signaling, such as *CaMs* and *CPKs* (Figure 7). *CaM* expression was up-regulated to comparable levels in *Xcc*-resistant and susceptible *B. napus* cultivars (Mamun et al., 2020), while *CaM* genes of *B. oleracea* were found to display variable expression patterns upon *Xcc* infection (Song et al., 2020). These differential phenomena in different studies could be explained by the multifunctionality of CaMs in plants in a wide range of biological processes, including plant development and abiotic stress responses (Wang et al., 2009; Parvin et al., 2012). Observed differences are also likely affected by the level of infection and host-isolate interaction studied (Song et al., 2020). CPKs are known to play myriad roles in immune signaling, such as those mediating cell death, pathogen restriction, and ROS burst (Gao et al., 2013). Recently, CPK3 was identified as a key regulator of both PTI and ETI required for resistance to *P. syringae* in *Arabidopsis*, and to play an important role in immune-associated cytoskeleton organization (Lu et al., 2020). In addition, the downstream transcription factor gene *WRKY11* was found to be up-regulated in the resistant *B. napus* mutant line upon *Xcc* infection. WRKY11 was shown to mediate Ca^2+^-dependent interactions with calmodulin and to act as a negative regulator of basal resistance to *P. syringae* in *Arabidopsis* (Park et al., 2005; Journot-Catalino et al., 2006).

Genes involved in hormone signaling displayed more variable and complex patterns of expression upon *Xcc* infection (Figure 7), which could be caused by the high degree of transcriptional crosstalk between the different phytohormone pathways. Strangely, no SA-related DEGs were detected among the two trait-correlated modules. This was somehow unexpected, given that SA was found to accumulate in a compatible *Xcc*-*B. napus* interaction as shown by Islam et al. (2019).

### Roles of secondary metabolites in *Xanthomonas campestris* pv. *campestris* resistance

Multiple studies revealed the importance of several metabolites in response to *Xcc*, such as glucosinolates, HAs, flavonoids, and phenolic contents. Such defensive metabolites particularly tend to accumulate in incompatible *Xcc*-Brassica interactions (Aires et al., 2011; Velasco et al., 2013; Barman et al., 2015; Islam et al., 2018). Our study also revealed transcriptional activation of similar metabolic and biosynthetic processes upon *Xcc* infection (Figure 5). Multiple DEGs involved in the flavonoid and phenylpropanoid pathways, however, were not found to be up-regulated in the resistant line ZS9m*Xcc*R-1, but rather in the susceptible line ZS9m*Xcc*S-1 at an early stage of infection (Supplementary Figure 6). This is in contrast with Islam et al. (2017), which shows enhanced expression of genes within diverse secondary metabolism pathways in an incompatible *Xcc*-*B. napus* interaction. Notably, two recent RNA-seq analyses on *Brassica-Xcc* interactions did not highlight the role of secondary metabolites in response to *Xcc* infection (Song et al., 2020; Sun et al., 2021). KEGG analysis of DEGs from the black and purple modules also did not reveal enriched metabolic pathways. Due to this inconsistency across different studies, the exact role of plant secondary metabolites in response to *Xcc* infection needs to be further studied in detail.

## Conclusion

Considerable efforts have been made to unravel the molecular mechanisms underlying *Xcc* resistance in Brassica crops in the last decade. In this study, we performed comparative transcriptome analyses on three *B. napus* lines with contrasting susceptibility using broader unbiased approaches to pinpoint pathways playing key functions in black rot resistance. Our results indicate that *B. napus* evades *Xcc* invasion and colonization by transcriptional activation of a multitude of genes involved in (i) early PTI-mediated defenses, including immune recognition and signaling, and (ii) in Ca^2+^ homeostasis and signaling. Together, this study provides novel insights into the molecular basis of black rot resistance in *B. napus* and may open new avenues in disease resistance breeding in Brassica crops.

## Data availability statement

The original contributions presented in this study are publicly available. This data can be found here: NCBI, PRJNA748871.

## Author contributions

SL, KB, and MS designed the research. LY performed the research and wrote the draft manuscript. CZ, ZB, and LLY assisted in experiments or data analysis. KB and SL edited the manuscript. All authors contributed to the article and approved the submitted version.

## Supplementary material

Supplementary Figure 1 Lesion lengths on 41 EMS-mutagenized *B. napus* lines at 8 days after *Xcc* inoculation.

Supplementary Figure 2 Disease symptoms on *B. napus* ZS9, ZS9m*Xcc*R-1, and ZS9m*Xcc*S-1 at 8 days after inoculation with diverse *Xcc* races/strains. Bars represent 10 mm.

Supplementary Figure 3 Bubble diagrams showing the distribution of GO enriched terms in ZS9m*Xcc*R-1 **(A)** and ZS9m*Xcc*S-1 **(B)**. Color bars and dot sizes refer to padj values of enriched GO terms and numbers of involved DEGs, respectively.

Supplementary Figure 4 Network topology analysis. **(A)** Topology and connectivity based on a multitude of soft thresholding powers. **(B)** Heatmaps of DEGs belonging to eight different modules. **(C)** Correlations between different modules and samples. Color bars represent negative (blue) and positive (red) correlations. **(D)** Expression of hub genes within black and purple modules at 0, 5, and 8 dpi, respectively.

Supplementary Figure 5 Correlation analysis of gene expression obtained by Q-RT-PCR and RNA-seq analysis.

Supplementary Figure 6 Heatmaps of DEGs involved in flavonoid **(A)** and phenylpropanoid **(B)** pathways.

Supplementary Data 1 R script for WGCNA analysis.
