# Supplementary figures and images for: Comparative transcriptome analysis of compatible and incompatible Brassica napus—Xanthomonas campestris interactions
Source: Front Plant Sci. 2022 Aug 29;13:960874. doi: 10.3389/fpls.2022.960874 (PMC9465390; doi:10.3389/fpls.2022.960874)

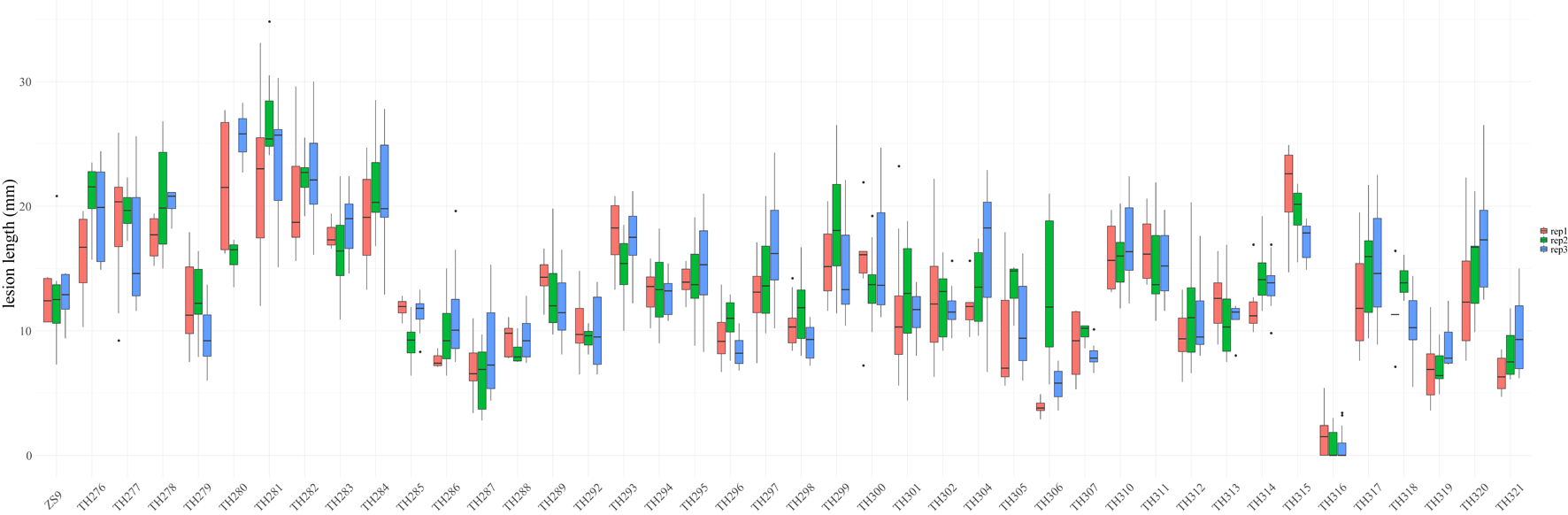

Supplement: Supplementary Figure 1 — Lesion lengths on 41 EMS-mutagenized B. napus lines at 8 days after Xcc inoculation. [file Image_1.TIF]

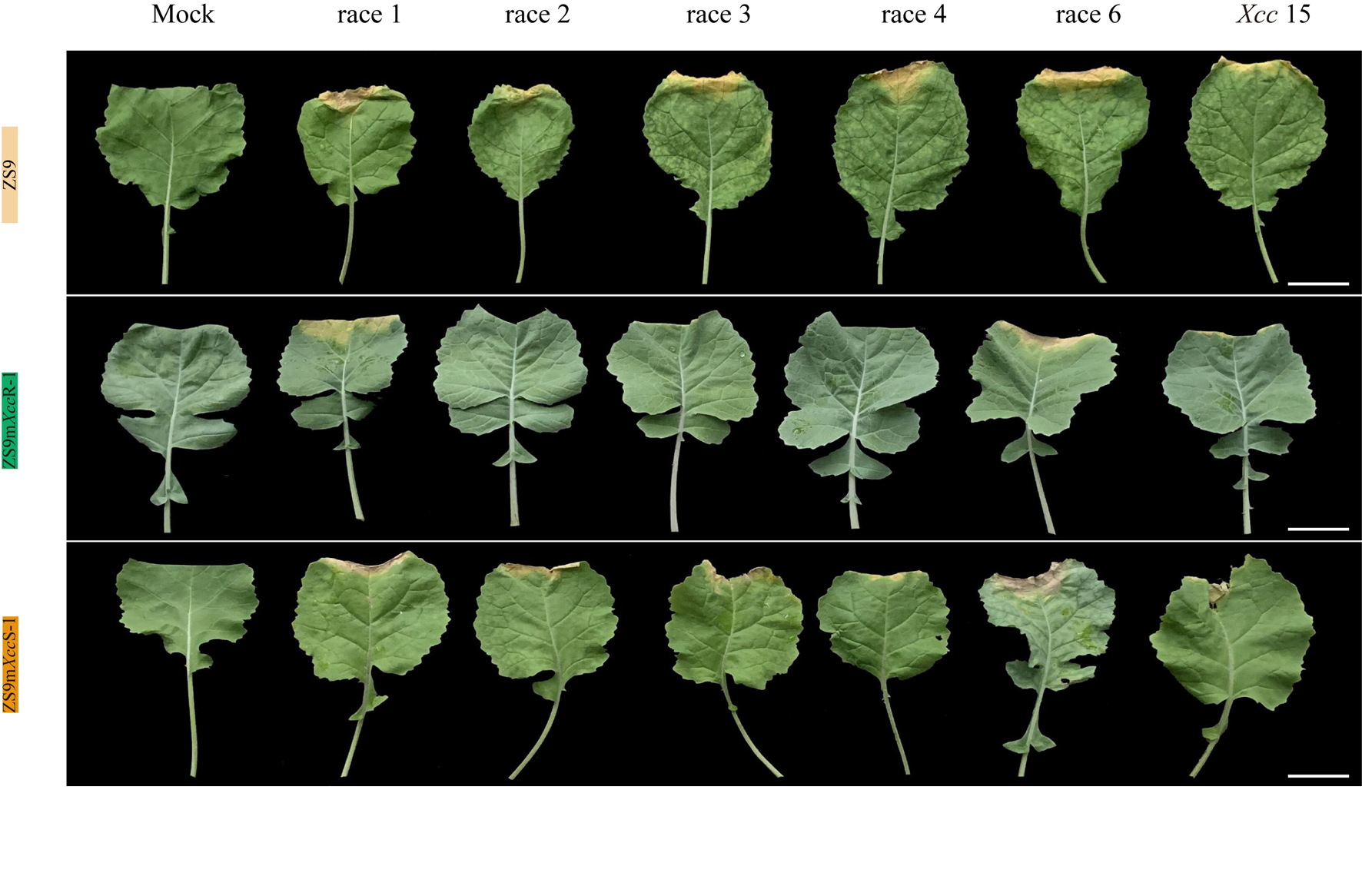

Supplement: Supplementary Figure 2 — Disease symptoms on B. napus ZS9, ZS9mXccR-1, and ZS9mXccS-1 at 8 days after inoculation with diverse Xcc races/strains. Bars represent 10 mm. [file Image_2.TIF]

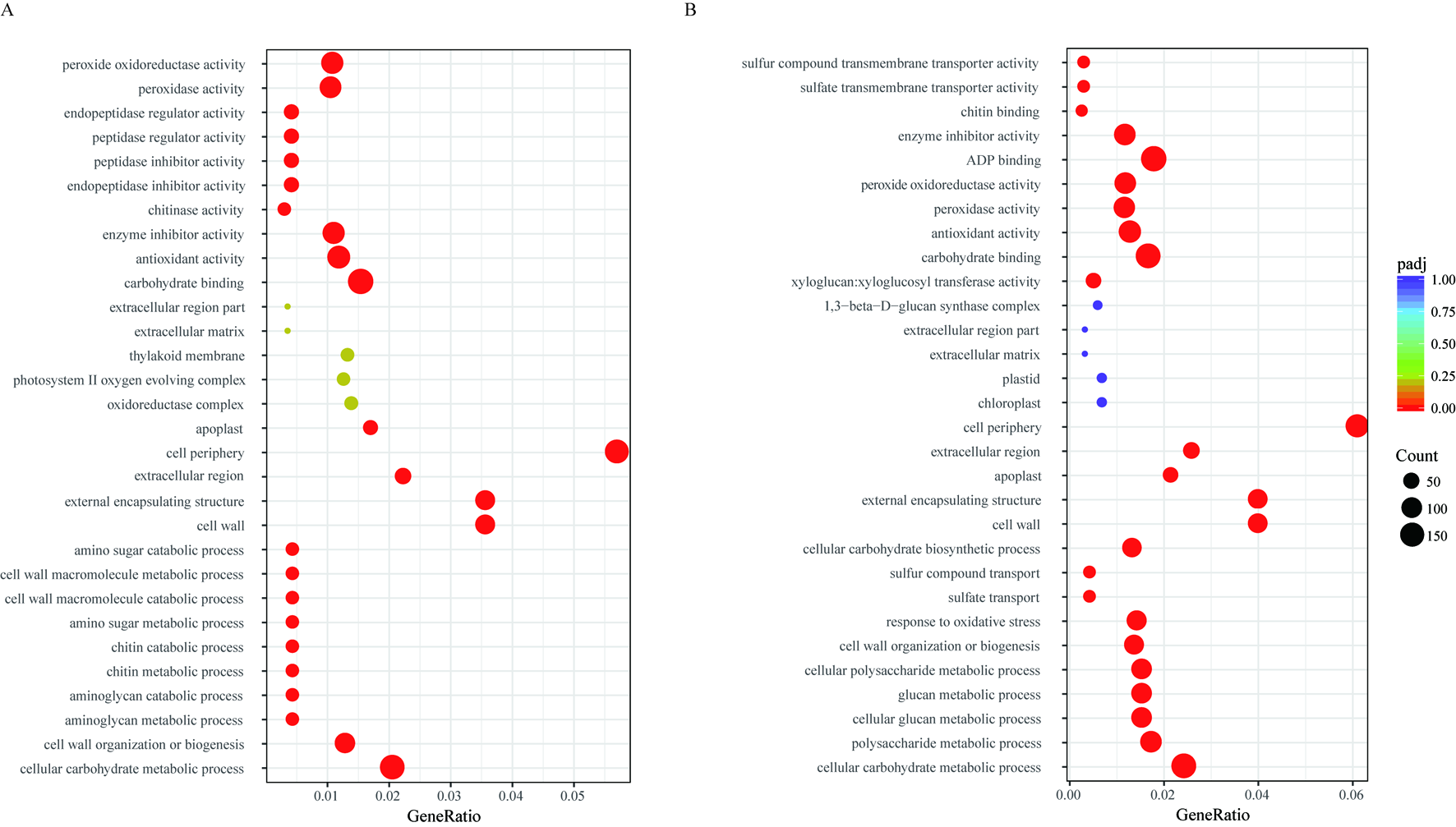

Supplement: Supplementary Figure 3 — Bubble diagrams showing the distribution of GO enriched terms in ZS9mXccR-1 (A) and ZS9mXccS-1 (B). Color bars and dot sizes refer to padj values of enriched GO terms and numbers of involved DEGs, respectively. [file Image_3.TIF]

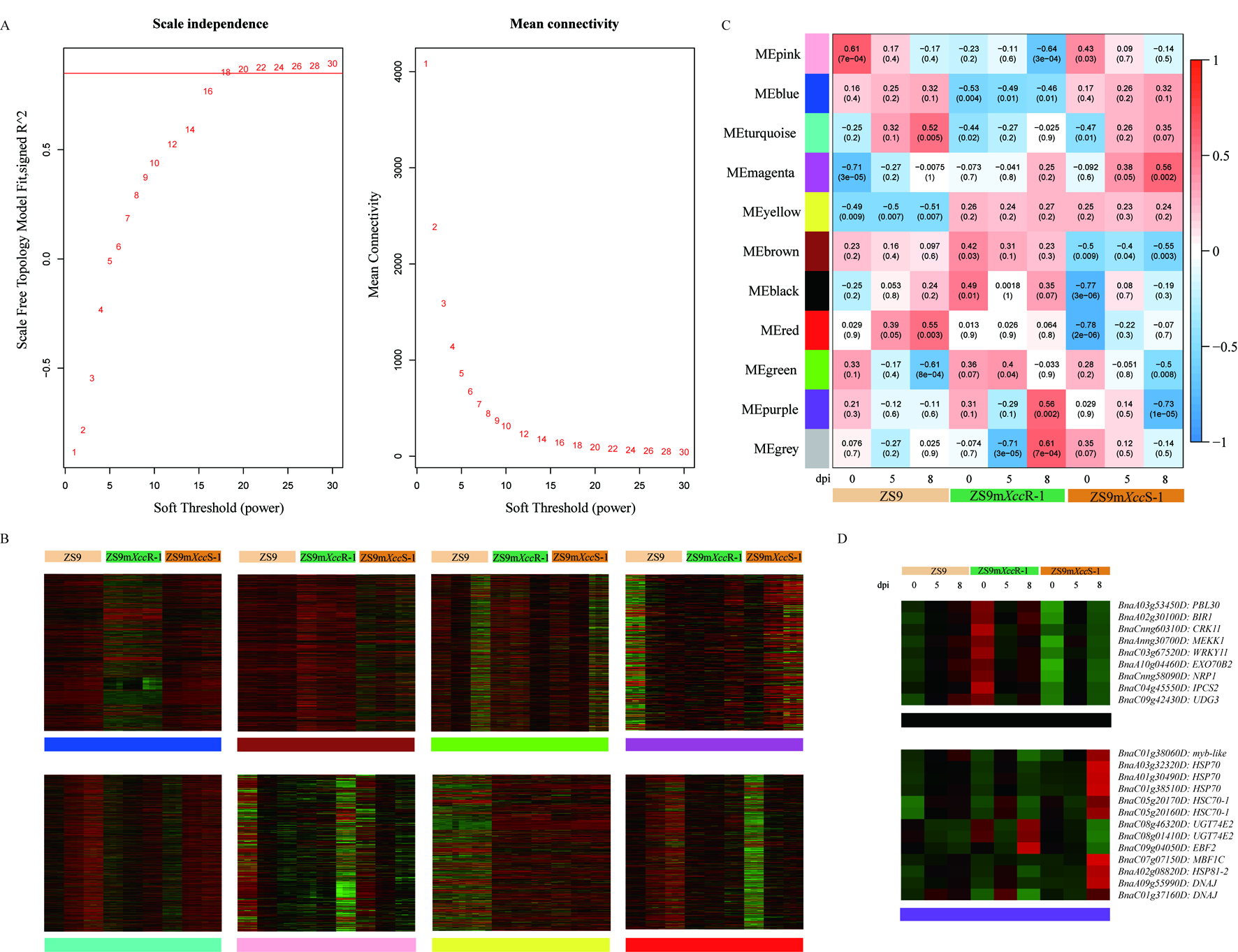

Supplement: Supplementary Figure 4 — Network topology analysis. (A) Topology and connectivity based on a multitude of soft thresholding powers. (B) Heatmaps of DEGs belonging to eight different modules. (C) Correlations between different modules and samples. Color bars represent negative (blue) and positive (red) correlations. (D) Expression of hub genes within black and purple modules at 0, 5, and 8 dpi, respectively. [file Image_4.TIF]

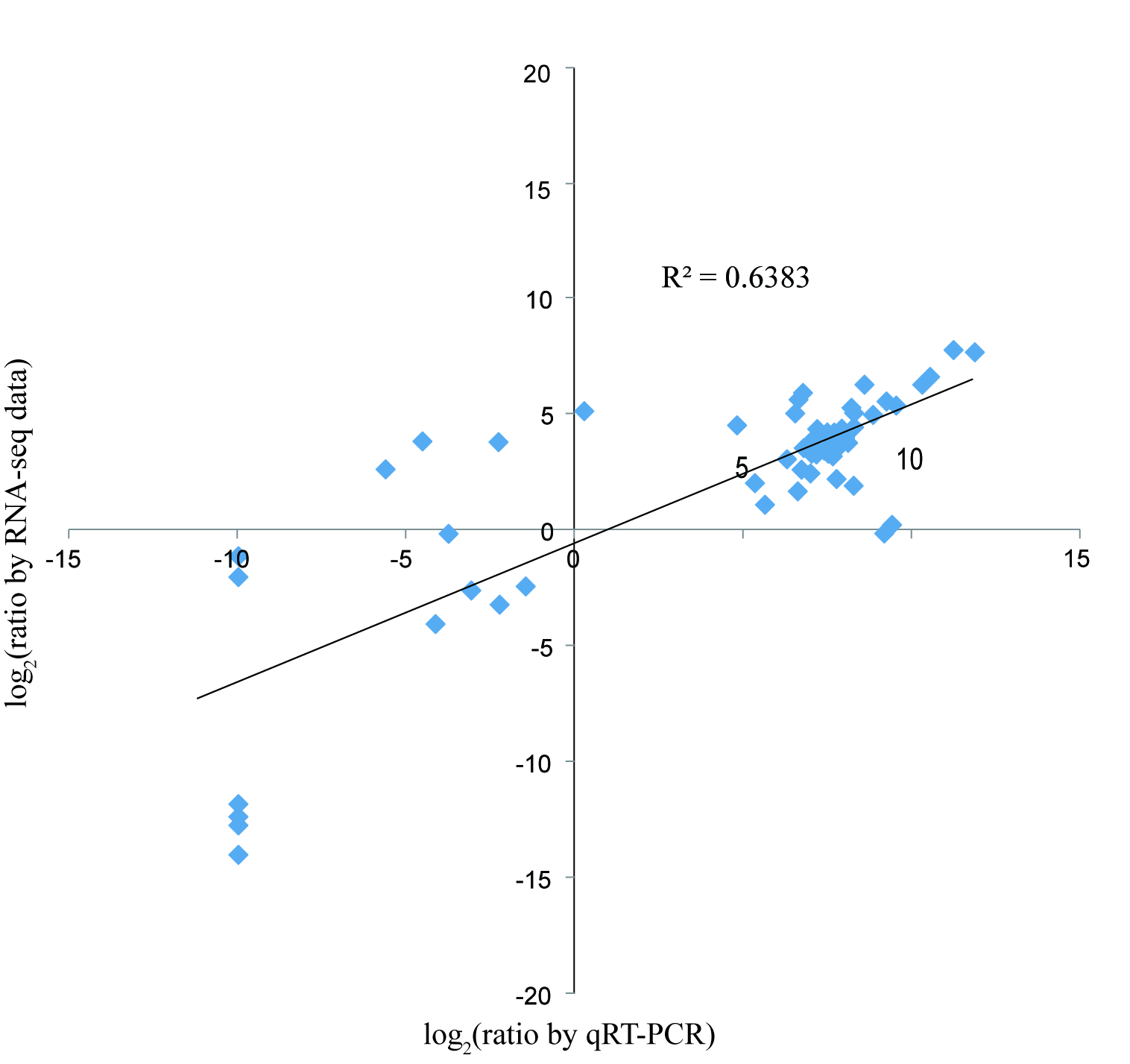

Supplement: Supplementary Figure 5 — Correlation analysis of gene expression obtained by Q-RT-PCR and RNA-seq analysis. [file Image_5.TIF]

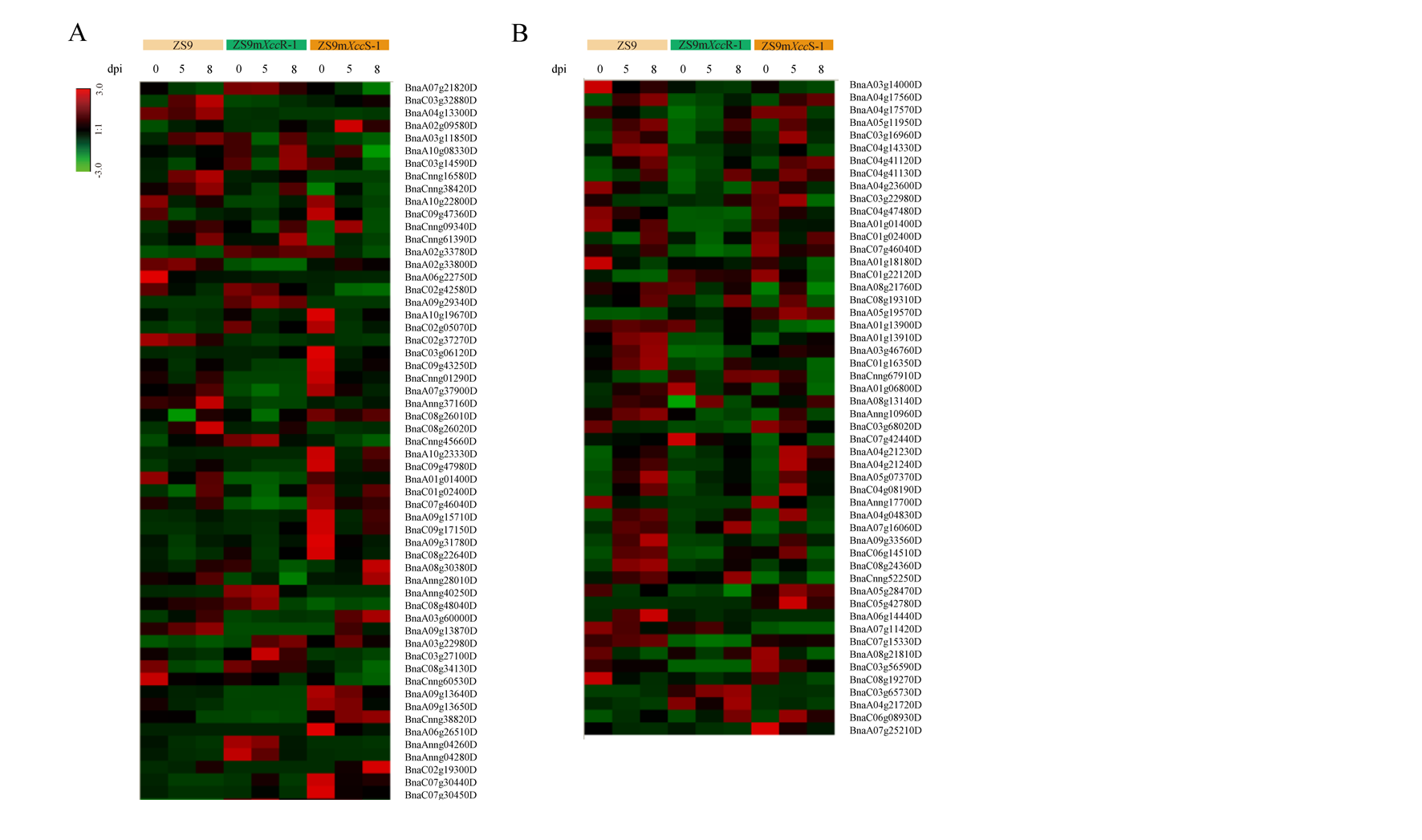

Supplement: Supplementary Figure 6 — Heatmaps of DEGs involved in flavonoid (A) and phenylpropanoid (B) pathways. [file Image_6.TIF]
