# Supplementary material for: Comparative transcriptome analysis of compatible and incompatible Brassica napus—Xanthomonas campestris interactions
Source: Front Plant Sci. 2022 Aug 29;13:960874. doi: 10.3389/fpls.2022.960874 (PMC9465390; doi:10.3389/fpls.2022.960874)
Supplement: Supplementary Data 1 — R script for WGCNA analysis. [file Data_Sheet_1.docx]

##if you have any questions, please do not hesitate to contact Li Yang, li2.yang@wur.nl or yangli2010119@163.com ##

library(WGCNA)

library(flashClust)

library(reshape2)

library(stringr)

options(stringsAsFactors = FALSE)

enableWGCNAThreads(nThreads=4)

datTraits = read.table("sample.info.txt",header=T)

data <-read.table("all.deg.sample.FPKM.txt",header=T)

keep=rowSums(data[,-1])>300

datatmp=data[keep,]

dim(datatmp)

data1=log(datatmp[,-1]+0.01)

rownames(data1)=paste(datatmp[,1])

datExpr = as.data.frame(t(data1))

nGenes = ncol(datExpr)

nSamples = nrow(datExpr)

gsg = goodSamplesGenes(datExpr, verbose = 3)

gsg$allOK

sampleTree = flashClust(dist(datExpr), method = "average")

pdf(file = "sampleClustering.pdf", width = 12, height = 9)

plot(sampleTree, main = "Sample clustering to detect outliers", sub="", xlab="", cex.lab = 1.5,cex.axis = 1.5, cex.main = 2)

dev.off()

powers = c(c(1:10), seq(from = 12, to=30, by=2))

sft = pickSoftThreshold(datExpr, powerVector = powers, verbose = 5)

pdf(file = "powers.pdf", width = 12, height = 9)

par(mfrow = c(1,2))

cex1 = 0.90

plot(sft$fitIndices[,1], -sign(sft$fitIndices[,3])*sft$fitIndices[,2],xlab="Soft Threshold (power)",ylab="Scale Free Topology Model Fit,signed R^2",type="n",main = paste("Scale independence"))

text(sft$fitIndices[,1], -sign(sft$fitIndices[,3])*sft$fitIndices[,2],labels=powers,cex=cex1,col="red")

abline(h=0.85,col="red")

plot(sft$fitIndices[,1], sft$fitIndices[,5],xlab="Soft Threshold (power)",ylab="Mean Connectivity", type="n",main = paste("Mean connectivity"))

text(sft$fitIndices[,1], sft$fitIndices[,5], labels=powers, cex=cex1,col="red")

dev.off()

k=softConnectivity(datExpr,power=18)

sizeGrWindow(10,5)

pdf("Histogram of k.pdf")

hist(k)

dev.off()

pdf("Check scale free topology.pdf")

par(mfrow=c(1,2))

hist(k)

scaleFreePlot(k,main="Check scale free topology\n")

dev.off()

net = blockwiseModules(datExpr, power = 18, maxBlockSize = 10000,

TOMType = "unsigned", minModuleSize = 50,

reassignThreshold = 0, mergeCutHeight = 0.25,

numericLabels = TRUE, pamRespectsDendro = FALSE,

saveTOMs = TRUE,

saveTOMFileBase = "RNA-seq-FPKM-TOM",

verbose = 3)

pdf(file="WGCNA_Module_Color.pdf",12,9)

mergedColors = labels2colors(net$colors)

plotDendroAndColors(net$dendrograms[[1]], mergedColors[net$blockGenes[[1]]],

"Module colors",

dendroLabels = FALSE, hang = 0.03,

addGuide = TRUE, guideHang = 0.05)

dev.off()

moduleLabels=net$colors

moduleColors=labels2colors(net$colors)

table(moduleColors)

gene_module <- data.frame(ID=colnames(datExpr), module=moduleColors)

gene_module = gene_module[order(gene_module$module),]

unique(moduleColors)

write.table(gene_module,file="gene_module.xls", sep="\t",quote=F,row.names=F)

module_blue = names(datExpr)[moduleColors=="blue"]

write.table(module_blue,file="module_blue.xls", sep="\t",quote=F,row.names=F,col.names=F)

module_yellow = names(datExpr)[moduleColors=="yellow"]

write.table(module_yellow,file="module_yellow.xls", sep="\t",quote=F,row.names=F,col.names=F)

module_green = names(datExpr)[moduleColors=="green"]

write.table(module_green,file="module_green.xls", sep="\t",quote=F,row.names=F,col.names=F)

module_turquoise = names(datExpr)[moduleColors=="turquoise"]

write.table(module_turquoise,file="module_turquoise.xls", sep="\t",quote=F,row.names=F,col.names=F)

module_red = names(datExpr)[moduleColors=="red"]

write.table(module_red,file="module_red.xls", sep="\t",quote=F,row.names=F,col.names=F)

module_brown = names(datExpr)[moduleColors=="brown"]

write.table(module_brown,file="module_brown.xls", sep="\t",quote=F,row.names=F,col.names=F)

module_magenta = names(datExpr)[moduleColors=="magenta"]

write.table(module_magenta,file="module_magenta.xls", sep="\t",quote=F,row.names=F,col.names=F)

module_pink = names(datExpr)[moduleColors=="pink"]

write.table(module_pink,file="module_pink.xls", sep="\t",quote=F,row.names=F,col.names=F)

module_black = names(datExpr)[moduleColors=="black"]

write.table(module_black,file="module_black.xls", sep="\t",quote=F,row.names=F,col.names=F)

module_grey = names(datExpr)[moduleColors=="grey"]

write.table(module_grey,file="module_grey.xls", sep="\t",quote=F,row.names=F,col.names=F)

module_grey = names(datExpr)[moduleColors=="purple"]

write.table(module_grey,file="module_purple.xls", sep="\t",quote=F,row.names=F,col.names=F)

which.module="blue"

pdf("module_blue.heatmap.pdf")

plotMat(

t(scale(datExpr[,moduleColors==which.module ]) ),

nrgcols=30,

rlabels=F,

rcols=which.module,

main=which.module,

cex.main=2

)

dev.off()

which.module="purple"

pdf("module_purple.heatmap.pdf")

plotMat(

t(scale(datExpr[,moduleColors==which.module ]) ),

nrgcols=30,

rlabels=F,

rcols=which.module,

main=which.module,

cex.main=2

)

dev.off()

which.module="yellow"

pdf("module_yellow.heatmap.pdf")

plotMat(

t(scale(datExpr[,moduleColors==which.module ]) ),

nrgcols=30,

rlabels=F,

rcols=which.module,

main=which.module,

cex.main=2

)

dev.off()

which.module="green"

pdf("module_green.heatmap.pdf")

plotMat(

t(scale(datExpr[,moduleColors==which.module ]) ),

nrgcols=30,

rlabels=F,

rcols=which.module,

main=which.module,

cex.main=2

)

dev.off()

which.module="turquoise"

pdf("module_turquoise.heatmap.pdf")

plotMat(

t(scale(datExpr[,moduleColors==which.module ]) ),

nrgcols=30,

rlabels=F,

rcols=which.module,

main=which.module,

cex.main=2

)

dev.off()

which.module="red"

pdf("module_red.heatmap.pdf")

plotMat(

t(scale(datExpr[,moduleColors==which.module ]) ),

nrgcols=30,

rlabels=F,

rcols=which.module,

main=which.module,

cex.main=2

)

dev.off()

which.module="brown"

pdf("module_brown.heatmap.pdf")

plotMat(

t(scale(datExpr[,moduleColors==which.module ]) ),

nrgcols=30,

rlabels=F,

rcols=which.module,

main=which.module,

cex.main=2

)

dev.off()

which.module="magenta"

pdf("module_magenta.heatmap.pdf")

plotMat(

t(scale(datExpr[,moduleColors==which.module ]) ),

nrgcols=30,

rlabels=F,

rcols=which.module,

main=which.module,

cex.main=2

)

dev.off()

which.module="pink"

pdf("module_pink.heatmap.pdf")

plotMat(

t(scale(datExpr[,moduleColors==which.module ]) ),

nrgcols=30,

rlabels=F,

rcols=which.module,

main=which.module,

cex.main=2

)

dev.off()

which.module="black"

pdf("module_black.heatmap.pdf")

plotMat(

t(scale(datExpr[,moduleColors==which.module ]) ),

nrgcols=30,

rlabels=F,

rcols=which.module,

main=which.module,

cex.main=2

)

dev.off()

which.module="grey"

pdf("module_grey.heatmap.pdf")

plotMat(

t(scale(datExpr[,moduleColors==which.module ]) ),

nrgcols=30,

rlabels=F,

rcols=which.module,

main=which.module,

cex.main=2

)

dev.off()

MEs = net$MEs

MEs_col = MEs

colnames(MEs_col) = paste0("ME", labels2colors(as.numeric(str_replace_all(colnames(MEs),"ME",""))))

MEs_col = orderMEs(MEs_col)

pdf("Eigengene adjacency heatmap.pdf")

plotEigengeneNetworks(MEs_col, "Eigengene adjacency heatmap",

marDendro = c(3,3,2,4),

marHeatmap = c(3,4,2,2), plotDendrograms = T,

xLabelsAngle = 90)

dev.off()

#form a data frame analogous to expression data that will hold the clinical traits.

rownames(datTraits) = datTraits$ID_REF

datTraits$ID_REF = NULL

table(rownames(datTraits)==rownames(datExpr)) #should return TRUE if datasets align correctly,otherwise your names are out of order

MEs0=moduleEigengenes(datExpr,moduleColors)$eigengenes

MEs = orderMEs(MEs0)

moduleTraitCor = cor(MEs, datTraits, use= "p")

moduleTraitPvalue = corPvalueStudent(moduleTraitCor, nSamples)

textMatrix= paste(signif(moduleTraitCor, 2), "\n(",signif(moduleTraitPvalue, 1), ")", sep= "")

dim(textMatrix)=dim(moduleTraitCor)

pdf(file="WGCNA_Module_Trait_Relationships.pdf",12,20)

par(mar=c(6,8.5,3,3))

labeledHeatmap(Matrix= moduleTraitCor,

xLabels= names(datTraits),

yLabels= names(MEs),

ySymbols= names(MEs),

colorLabels= FALSE,

colors= blueWhiteRed(50),

textMatrix= textMatrix,

setStdMargins= FALSE,

cex.text= 0.5,

zlim= c(-1,1),

main= paste("Module-trait relationships"))

dev.off()

which.trait <- "ZS9mXccR-1_8"

moduleTraitCor[, which.trait]

moduleColors = labels2colors(net$colors)

which.trait <- "ZS9mXccR-1_8"

y <- datTraits[, which.trait]

GS <- as.numeric(cor(y ,datExpr, use="p"))

GeneSignificance <- GS

ModuleSignificance <- tapply(GeneSignificance,moduleColors, mean, na.rm=T)

#pdf("GeneSignificance.across.Modules.of.ZS9mXccR-1_8.pdf")

plotModuleSignificance(GeneSignificance, moduleColors,ylim=c(-1,1),main="Gene significance across modules",ylab = "Gene Significance")

dev.off()

modNames = substring(names(MEs), 3)

geneModuleMembership = as.data.frame(cor(datExpr, MEs, use = "p"))

MMPvalue = as.data.frame(corPvalueStudent(as.matrix(geneModuleMembership), nSamples))

names(geneModuleMembership) = paste("MM", modNames, sep="")

names(MMPvalue) = paste("p.MM", modNames, sep="")

ZS9mXccR-1_8 = as.data.frame(datTraits[,6])

names(ZS9mXccR-1_8) = "ZS9mXccR-1_8"

geneTraitSignificance = as.data.frame(cor(datExpr, ZS9mXccR-1_8, use = "p"))

GSPvalue = as.data.frame(corPvalueStudent(as.matrix(geneTraitSignificance), nSamples))

names(geneTraitSignificance) = paste("GS.", names(ZS9mXccR-1_8), sep="")

names(GSPvalue) = paste("p.GS.", names(ZS9mXccR-1_8), sep="")

module="purple"

column = match(module, modNames)

moduleGenes = moduleColors==module

sizeGrWindow(7, 7)

par(mfrow = c(1,1))

pdf("Module.purple.vs.geneforZS9mXccR-1_8.pdf")

verboseScatterplot(abs(geneModuleMembership[moduleGenes, column]),

abs(geneTraitSignificance[moduleGenes, 1]),

xlab = paste("Module Membership in", module, "module"),

ylab = "Gene significance for ZS9mXccR-1_8",

main = paste("Module membership vs. gene significance\n"),

cex.main = 1.2, cex.lab = 1.2, cex.axis = 1.2, col = module)

dev.off()

which.trait <- "ZS9mXccS-1_8"

moduleTraitCor[, which.trait]

moduleColors = labels2colors(net$colors)

which.trait <- "ZS9mXccS-1_8"

y <- datTraits[, which.trait]

GS <- as.numeric(cor(y ,datExpr, use="p"))

GeneSignificance <- GS

ModuleSignificance <- tapply(GeneSignificance,moduleColors, mean, na.rm=T)

pdf("GeneSignificance.across.Modules.of.ZS9mXccS-1_8.pdf")

plotModuleSignificance(GeneSignificance, moduleColors,ylim=c(-1,1),main="Gene significance across modules",ylab = "Gene Significance")

dev.off()

modNames = substring(names(MEs), 3)

geneModuleMembership = as.data.frame(cor(datExpr, MEs, use = "p"))

MMPvalue = as.data.frame(corPvalueStudent(as.matrix(geneModuleMembership), nSamples))

names(geneModuleMembership) = paste("MM", modNames, sep="")

names(MMPvalue) = paste("p.MM", modNames, sep="")

ZS9mXccS-1_8 = as.data.frame(datTraits[,9])

names(ZS9mXccS-1_8) = "ZS9mXccS-1_8"

geneTraitSignificance = as.data.frame(cor(datExpr, ZS9mXccS-1_8, use = "p"))

GSPvalue = as.data.frame(corPvalueStudent(as.matrix(geneTraitSignificance), nSamples))

names(geneTraitSignificance) = paste("GS.", names(ZS9mXccS-1_8), sep="")

names(GSPvalue) = paste("p.GS.", names(ZS9mXccS-1_8), sep="")

module="magenta"

column = match(module, modNames)

moduleGenes = moduleColors==module

sizeGrWindow(7, 7)

par(mfrow = c(1,1))

pdf("Module.magenta.vs.geneforZS9mXccS-1_8.pdf")

verboseScatterplot(abs(geneModuleMembership[moduleGenes, column]),

abs(geneTraitSignificance[moduleGenes, 1]),

xlab = paste("Module Membership in", module, "module"),

ylab = "Gene significance for ZS9mXccS-1_8",

main = paste("Module membership vs. gene significance\n"),

cex.main = 1.2, cex.lab = 1.2, cex.axis = 1.2, col = module)

dev.off()

which.trait <- "ZS9mXccR-1_0"

moduleTraitCor[, which.trait]

moduleColors = labels2colors(net$colors)

which.trait <- "ZS9mXccR-1_0"

y <- datTraits[, which.trait]

GS <- as.numeric(cor(y ,datExpr, use="p"))

GeneSignificance <- GS

ModuleSignificance <- tapply(GeneSignificance,moduleColors, mean, na.rm=T)

pdf("GeneSignificance.across.Modules.of.ZS9mXccR-1_0.pdf")

plotModuleSignificance(GeneSignificance, moduleColors,ylim=c(-1,1),main="Gene significance across modules",ylab = "Gene Significance")

dev.off()

modNames = substring(names(MEs), 3)

geneModuleMembership = as.data.frame(cor(datExpr, MEs, use = "p"))

MMPvalue = as.data.frame(corPvalueStudent(as.matrix(geneModuleMembership), nSamples))

names(geneModuleMembership) = paste("MM", modNames, sep="")

names(MMPvalue) = paste("p.MM", modNames, sep="")

ZS9mXccR-1_0 = as.data.frame(datTraits[,4])

names(ZS9mXccR-1_0) = "ZS9mXccR-1_0"

geneTraitSignificance = as.data.frame(cor(datExpr, ZS9mXccR-1_0, use = "p"))

GSPvalue = as.data.frame(corPvalueStudent(as.matrix(geneTraitSignificance), nSamples))

names(geneTraitSignificance) = paste("GS.", names(ZS9mXccR-1_0), sep="")

names(GSPvalue) = paste("p.GS.", names(ZS9mXccR-1_0), sep="")

module="black"

column = match(module, modNames)

moduleGenes = moduleColors==module

sizeGrWindow(7, 7)

par(mfrow = c(1,1))

pdf("Module.black.vs.geneforZS9mXccR-1_0.pdf")

verboseScatterplot(abs(geneModuleMembership[moduleGenes, column]),

abs(geneTraitSignificance[moduleGenes, 1]),

xlab = paste("Module Membership in", module, "module"),

ylab = "Gene significance for ZS9mXccR-1_0",

main = paste("Module membership vs. gene significance\n"),

cex.main = 1.2, cex.lab = 1.2, cex.axis = 1.2, col = module)

dev.off()

geneTree = net$dendrograms[[1]]

moduleColors = labels2colors(net$colors)

dissTOM = 1 - TOMsimilarityFromExpr(datExpr,power = 18)

nSelect = 1000

set.seed(10)

select = sample(nGenes, size = nSelect)

selectTOM = dissTOM[select, select]

selectTree = hclust(as.dist(selectTOM), method = "average")

selectColors = moduleColors[select]

sizeGrWindow(9,9)

plotDiss = selectTOM^7

diag(plotDiss) = NA

pdf("Network heatmap plot.pdf")

TOMplot(plotDiss, selectTree, selectColors, main = "Network heatmap plot, selected genes")

dev.off()

library(gplots)

myheatcol = colorpanel(250,'red',"orange",'lemonchiffon')

TOMplot(plotDiss, selectTree, selectColors, main = "Network heatmap plot, selected genes",col=myheatcol)

TOM = 1-dissTOM

# Read in the annotation file

# annot = read.csv(file = "GeneAnnotation.csv");

# Select modules

modules = c("purple")

# Select module probes

probes = names(datExpr)

inModule = is.finite(match(moduleColors, modules))

modProbes = probes[inModule]

#modGenes = annot$gene_symbol[match(modProbes, annot$substanceBXH)]

# Select the corresponding Topological Overlap

modTOM = TOM[inModule, inModule]

dimnames(modTOM) = list(modProbes, modProbes)

# Export to VisANT

vis = exportNetworkToVisANT(modTOM,

file = paste("VisANTInput-", modules, ".txt", sep=""),

weighted = TRUE,

threshold = 0)

modules = c("black")

# Select module probes

probes = names(datExpr)

inModule = is.finite(match(moduleColors, modules))

modProbes = probes[inModule]

#modGenes = annot$gene_symbol[match(modProbes, annot$substanceBXH)]

# Select the corresponding Topological Overlap

modTOM = TOM[inModule, inModule]

dimnames(modTOM) = list(modProbes, modProbes)

# Export to VisANT

vis = exportNetworkToVisANT(modTOM,

file = paste("VisANTInput-", modules, ".txt", sep=""),

weighted = TRUE,

threshold = 0)

modules = c("magenta")

# Select module probes

probes = names(datExpr)

inModule = is.finite(match(moduleColors, modules))

modProbes = probes[inModule]

#modGenes = annot$gene_symbol[match(modProbes, annot$substanceBXH)]

# Select the corresponding Topological Overlap

modTOM = TOM[inModule, inModule]

dimnames(modTOM) = list(modProbes, modProbes)

# Export to VisANT

vis = exportNetworkToVisANT(modTOM,

file = paste("VisANTInput-", modules, ".txt", sep=""),

weighted = TRUE,

threshold = 0)

##hub gene in purple module (ZS9mXccR-1_8)

#Calculate Intramodular connectivity

moduleColors <- labels2colors(net$colors)

connet=abs(cor(datExpr,use="p"))^6

Alldegrees1=intramodularConnectivity(connet, moduleColors)

head(Alldegrees1)

#calculate relationship between gene significance and intramodular connectivity

which.module="purple"

ZS9mXccR-1_8 = as.data.frame(datTraits[,6]) # change specific

names(ZS9mXccR-1_8) = "ZS9mXccR-1_8"

GS1 = as.numeric(cor(ZS9mXccR-1_8,datExpr, use="p"))

GeneSignificance=abs(GS1)

colorlevels=unique(moduleColors)

sizeGrWindow(9,6)

#pdf("relationship between gene significance and intramodular connectivity.pdf")

par(mfrow=c(2,as.integer(0.5+length(colorlevels)/2)))

par(mar = c(4,5,3,1))

for (i in c(1:length(colorlevels)))

{

whichmodule=colorlevels[[i]]

restrict1 = (moduleColors == whichmodule)

verboseScatterplot(Alldegrees1$kWithin[restrict1],

GeneSignificance[restrict1], col=moduleColors[restrict1],

main=whichmodule,

xlab = "Connectivity", ylab = "Gene Significance", abline = TRUE)

}

dev.off()

#Calculate the connectivity of all genes in the module.Screen the hub genes.

#abs(GS1)> 0.9 (could be adjusted depend on your data)

#abs(datKME$MM.black)>0.8 (at least more than 0.8)

#Generalizing intramodular connectivity for all genes on the array

datKME=signedKME(datExpr, MEs, outputColumnName="MM.")

head(datKME)

write.csv(datKME,file = "datKME.csv")

#Finding genes with high gene significance and high intramodular connectivity in interesting modules

FilterGenes= abs(GS1)> 0.8 & abs(datKME$MM.purple) > 0.8

table(FilterGenes)

x = datKME[(GS1>0.8 & datKME$MM.purple >0.8),]

View(x)

write.csv(x, file="module_purple_hub_gene_GS0.8.csv")

#Calculate Intramodular connectivity

moduleColors <- labels2colors(net$colors)

connet=abs(cor(datExpr,use="p"))^6

Alldegrees1=intramodularConnectivity(connet, moduleColors)

head(Alldegrees1)

#calculate relationship between gene significance and intramodular connectivity

which.module="magenta"

ZS9mXccS-1_8 = as.data.frame(datTraits[,9]) # change specific

names(ZS9mXccS-1_8) = "ZS9mXccS-1_8"

GS1 = as.numeric(cor(ZS9mXccS-1_8,datExpr, use="p"))

GeneSignificance=abs(GS1)

colorlevels=unique(moduleColors)

sizeGrWindow(9,6)

pdf("relationship between gene significance and intramodular connectivity.pdf")

par(mfrow=c(2,as.integer(0.5+length(colorlevels)/2)))

par(mar = c(4,5,3,1))

for (i in c(1:length(colorlevels)))

{

whichmodule=colorlevels[[i]]

restrict1 = (moduleColors == whichmodule)

verboseScatterplot(Alldegrees1$kWithin[restrict1],

GeneSignificance[restrict1], col=moduleColors[restrict1],

main=whichmodule,

xlab = "Connectivity", ylab = "Gene Significance", abline = TRUE)

}

dev.off()

#Calculate the connectivity of all genes in the module.Screen the hub genes.

#abs(GS1)> 0.9 (could be adjusted depend on your data)

#abs(datKME$MM.black)>0.8 (at least more than 0.8)

#Generalizing intramodular connectivity for all genes on the array

datKME=signedKME(datExpr, MEs, outputColumnName="MM.")

head(datKME)

write.csv(datKME,file = "datKME.csv")

#Finding genes with high gene significance and high intramodular connectivity in interesting modules

FilterGenes= abs(GS1)> 0.8 & abs(datKME$MM.magenta) > 0.8

table(FilterGenes)

x = datKME[(GS1>0.8 & datKME$MM.magenta >0.8),]

View(x)

write.csv(x, file="module_magenta_hub_gene_GS0.8.csv")

#Calculate Intramodular connectivity

moduleColors <- labels2colors(net$colors)

connet=abs(cor(datExpr,use="p"))^6

Alldegrees1=intramodularConnectivity(connet, moduleColors)

head(Alldegrees1)

#calculate relationship between gene significance and intramodular connectivity

which.module="black"

ZS9mXccR-1_0 = as.data.frame(datTraits[,4]) # change specific

names(ZS9mXccR-1_0) = "ZS9mXccR-1_0"

GS1 = as.numeric(cor(ZS9mXccR-1_0,datExpr, use="p"))

GeneSignificance=abs(GS1)

colorlevels=unique(moduleColors)

sizeGrWindow(9,6)

pdf("relationship between gene significance and intramodular connectivity.pdf")

par(mfrow=c(2,as.integer(0.5+length(colorlevels)/2)))

par(mar = c(4,5,3,1))

for (i in c(1:length(colorlevels)))

{

whichmodule=colorlevels[[i]]

restrict1 = (moduleColors == whichmodule)

verboseScatterplot(Alldegrees1$kWithin[restrict1],

GeneSignificance[restrict1], col=moduleColors[restrict1],

main=whichmodule,

xlab = "Connectivity", ylab = "Gene Significance", abline = TRUE)

}

dev.off()

#Calculate the connectivity of all genes in the module.Screen the hub genes.

#abs(GS1)> 0.9 (could be adjusted depend on your data)

#abs(datKME$MM.black)>0.8 (at least more than 0.8)

#Generalizing intramodular connectivity for all genes on the array

datKME=signedKME(datExpr, MEs, outputColumnName="MM.")

head(datKME)

write.csv(datKME,file = "datKME.csv")

#Finding genes with high gene significance and high intramodular connectivity in interesting modules

FilterGenes= abs(GS1)> 0.8 & abs(datKME$MM.black) > 0.8

table(FilterGenes)

x = datKME[(GS1>0.8 & datKME$MM.black >0.8),]

View(x)

write.csv(x, file="module_black_hub_gene_GS0.8.csv")

FilterGenes= abs(GS1)> 0.75 & abs(datKME$MM.black) > 0.8

table(FilterGenes)

x = datKME[(GS1>0.75 & datKME$MM.black >0.8),]

View(x)

write.csv(x, file="module_black_hub_gene_GS0.75.csv")
